# In Vitro Bioeffects of Polyelectrolyte Multilayer Microcapsules Post-Loaded with Water-Soluble Cationic Photosensitizer

**DOI:** 10.3390/pharmaceutics12070610

**Published:** 2020-06-30

**Authors:** Alexey V. Ermakov, Roman A. Verkhovskii, Irina V. Babushkina, Daria B. Trushina, Olga A. Inozemtseva, Evgeny A. Lukyanets, Vladimir J. Ulyanov, Dmitry A. Gorin, Sergei Belyakov, Maria N. Antipina

**Affiliations:** 1 Institute of Materials Research and Engineering, Agency for Science, Technology and Research (A*STAR), 2 Fusionopolis Way, Innovis, #08-03, Singapore 138634, Singapore; ermakov.ssu@gmail.com; 2 Saratov State University, Astrakhanskaya St 83, 410012 Saratov, Russia; r.a.verhovskiy@mail.ru (R.A.V.); inozemtsevaoa@mail.ru (O.A.I.); 3 I.M. Sechenov First Moscow State Medical University, Bol’shaya Pirogovskaya St 19c1, 119146 Moscow, Russia; trushina.d@mail.ru; 4 Yuri Gagarin State Technical University of Saratov, Politehnicheskaya St 77, 410054 Saratov, Russia; 5 Institute of Traumatology and Orthopedics, Saratov Medical State University, Chernyshevskaya St 148, 410002 Saratov, Russia; 10051968@mail.ru (I.V.B.); v.u.ulyanov@gmail.com (V.J.U.); 6 A.V. Shubnikov Institute of Crystallography of Federal Scientific Research Centre “Crystallography and Photonics” of Russian Academy of Sciences, Leninskiy Prospekt 59, 119333 Moscow, Russia; 7 Organic Intermediates and Dyes Institute, B. Sadovaya St ¼, 101999 Moscow, Russia; rmeluk@niopik.ru; 8 Skolkovo Institute of Science and Technology, Bolshoy Blvd 30, bld. 1, 121205 Moscow, Russia; D.Gorin@skoltech.ru; 9 Theracross Technologies Pte Ltd, 250p Pasir Panjang Rd, Singapore 117452, Singapore; belyakov_s@yahoo.com

**Keywords:** polyelectrolyte multilayer microcapsules, photodynamic therapy, Cholosens, post-loading, high-temperature treatment, encapsulation efficacy

## Abstract

Microencapsulation and targeted delivery of cytotoxic and antibacterial agents of photodynamic therapy (PDT) improve the treatment outcomes for infectious diseases and cancer. In many cases, the loss of activity, poor encapsulation efficiency, and inadequate drug dosing hamper the success of this strategy. Therefore, the development of novel and reliable microencapsulated drug formulations granting high efficacy is of paramount importance. Here we report the in vitro delivery of a water-soluble cationic PDT drug, zinc phthalocyanine choline derivative (Cholosens), by biodegradable microcapsules assembled from dextran sulfate (DS) and poly-l-arginine (PArg). A photosensitizer was loaded in pre-formed [DS/PArg]_4_ hollow microcapsules with or without exposure to heat. Loading efficacy and drug release were quantitatively studied depending on the capsule concentration to emphasize the interactions between the DS/PArg multilayer network and Cholosens. The loading data were used to determine the dosage for heated and intact capsules to measure their PDT activity in vitro. The capsules were tested using human cervical adenocarcinoma (HeLa) and normal human dermal fibroblast (NHDF) cell lines, and two bacterial strains, Gram-positive *Staphylococcus aureus* and Gram-negative *Escherichia coli*. Our results provide compelling evidence that encapsulated forms of Cholosens are efficient as PDT drugs for both eukaryotic cells and bacteria at specified capsule-to-cell ratios.

## 1. Introduction

Photodynamic therapy (PDT) is one of the modern methods for the treatment of cancer and infectious diseases that hold the promise of selective erasing of the pathologic area [1,2,3]. PDT is performed through the delivery of a photosensitizing agent with negligible dark toxicity to a diseased tissue, followed by excitation of the drug with the light of an appropriate wavelength. In response to light, the photosensitizer produces free radicals and/or reactive oxygen species that kill the target pathogenic or cancer cells [4,5]. Photosensitizer molecules with strong absorption peaks in the far-red or near-infrared regions are preferential for use in PDT because they allow the treatment of deeper tissues due to deeper penetration of the activation energy in comparison with the drugs absorbing at shorter wavelengths. The efficacy of PDT could be increased further by targeting the affected area with a delivery platform, which helps to improve the site-specificity and bioavailability of the PDT drug, thereby producing minimal impacts of drug activation on the surrounding healthy tissues [6,7,8,9,10,11].

Several organic and inorganic particle formulations of the PDT drugs for cancer treatment have shown superiority over the respective free therapeutics [6,11,12,13]. A clear preference based on a higher efficacy belongs to nanomaterials capable of passively targeting tumors via the enhanced permeability and retention (EPR) effect. However, multiple deficiencies of nanomaterials related to their inherited properties, e.g., structural instability, or their systemic, long-term, and dose-dependent toxicities [11], are promoting the development of safer alternatives. Micron or submicron-sized polymeric multilayer capsules (PMC) assembled on CaCO_3_ vaterite sacrificial templates via alternate adsorption of biodegradable polyelectrolytes represent the system of choice for the delivery of PDT drugs, owing to their long-term structural stability and low toxicity [14,15]. PMC have been developed as a universal delivery platform for many essential biologically active molecules, such as growth factors [16,17,18], antigens [19], unspecific components of the immune system [20], enzymes [21,22,23,24], DNA [25,26], RNA [27,28,29], and anticancer drugs [30,31], providing multiple options for targeting and controlled release by remote and local physical, chemical, and biological stimuli [32,33]. Advancements in the miniaturization of vaterite templates and their respectively assembled PMC [34,35,36] have eased a long-held concern about the ability of such capsules to penetrate tissues and cells for delivery of drugs via intravenous injections. PMC with the size of ~250 nm were internalized by macrophages and epithelial cells of the lungs and liver with efficacy higher than 75% when administered in mice through an injection into the tail vein [31].

There are two widely accepted classic ways to encapsulate a molecular payload in polymeric multilayers. Depending on the succeeding order of the capsule assembly and loading, they are respectively called pre-loading and post-loading. Pre-loading involves absorption of the molecules of interest by templates (e.g., porous vaterite or mesoporous silica particles) followed by the capsule assembly, with subsequent template dissolution. Post-loading comprises molecular diffusion of the drug in the hollow PMC upon immersion into a solution of the payload [37,38]. The efficacy of each loading method strongly depends on the permeability of the polymeric multilayer network by the payload molecules, which can be altered by varying the pH of the continuous phase [39,40,41] or by the exposure to heat [42,43,44].

Until recently, the polyelectrolyte multilayer network was considered a membrane that is not permeable for large molecules with a molecular weight exceeding 1 kDa [45]. According to this concept, free diffusion of smaller molecules was thus unrestricted. As such, the encapsulation of low molecular weight, water-soluble compounds has not been attempted using the classic loading methods. In a series of publications [31,36,46], we extended this concept by bringing into consideration the ionic charge compensations occurring within the multilayer infrastructure [47]. There are two types of charge compensations in the PMC network: one that is formed by the interaction of the multilayer polyelectrolytes (we define it as “intraneous” compensation); the other one is the “extraneous” compensation held by the original counterions coming with the individual polyelectrolytes before forming the PMC. These are small oppositely-charged ions of the water phase, such as H^+^, Na^+^, OH^−^, Cl^−^, etc. We envisioned that in such complex, intermixed ionic surroundings, the payload molecules of ionic nature would compete with the extraneous ions for respective resident ions in the polyelectrolyte network, thereby partially getting trapped in the capsule membrane [46]. Consistent with this assumption, we successfully applied a classic post-loading method to encapsulate low molecular weight water-soluble compounds in PMC [31,46], providing experimental evidence that a certain portion of the ionic payload gets associated with the polyelectrolyte multilayer wall, rather than arrives into the capsule interior [46].

High-temperature treatment of post-loaded PMC increases the number of polymer groups involved in the intraneous charge compensation, leading to the following three consequences: (1) tightening up the pores in the polyelectrolyte multilayer network and thus sealing the capsule; (2) capsule shrinking; (3) displacement and loss of some amount of payload associated with the polyelectrolyte multilayer wall via the extraneous charge compensation. Elsewhere, we have studied the physical-chemical aspects of the temperature effect on PMC, including detailed comparative research of the loading efficacy for rhodamine B for heated and intact capsules made of different polyelectrolyte pairs [36,46]. In addition, we have demonstrated a similar cell internalization rate in vitro for heated and intact capsules [31]; however, a complete dataset revealing the influence of temperature on the therapeutic efficacy of PMC post-loaded with low molecular weight water-soluble drugs is still missing.

**Figure 1:**
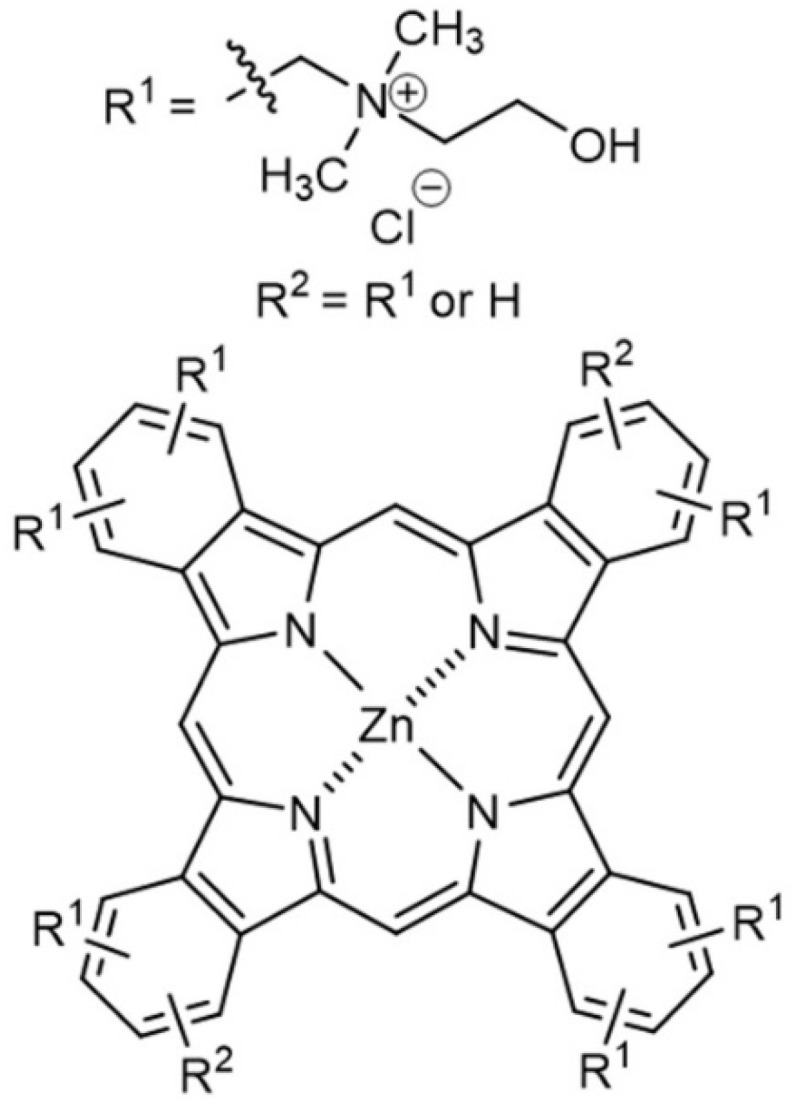
Chemical structure of Cholosens.

Small-to-medium molecular size PDT drugs stable to heating up to 70–90 °C are excellent models with which to discover the potential of PMC as a delivery platform for therapeutics encapsulated via the post-loading method. In this work, we used the phthalocyanine-based dye, octakis{methylene[N-(2hydroxyethyl)-N,N-dimethylammonium]}chloride zinc phthalocyanine, M = 1610 g/mol (Figure 1), which is currently marketed under the trade name Cholosens in Europe, as a PDT drug for oncology and infectious diseases in dentistry, otorhinolaryngology, gynecology, urology, and surgeries of various etiology [48,49,50,51,52,53]. Cholosens has an absorption peak at 680 nm. Light irradiation at this wavelength causes the generation of singlet oxygen from Cholosens molecule with a high quantum yield of 100 J/cm^2^ [54]. A recent study revealed the photobiological properties of non-encapsulated phthalocyanine photosensitizers, including Cholosens [55]. The current work aims to determine the efficacy of dextran sulfate (DS) and poly-l-arginine (PArg) PMC ([DS/PAgr]_4_) capsules loaded with Cholosens for the PDT treatment on eukaryotic cells and bacteria studied in vitro. Human adenocarcinoma (HeLa) cells and normal human dermal fibroblasts (NHDF) were used as corresponding models of cancer and normal eukaryotic cells. The antimicrobial PDT effect was studied on Gram-positive and Gram-negative strains, *Staphylococcus aureus* and *Escherichia coli*, respectively. In our work, we post-loaded the capsules with Cholosens with and without exposure to high temperature (80 °C for 60 min), while also varying the capsule concentration to get a better understanding of ionic interactions between the DS/PArg multilayer capsules and Cholosens. PMC loading was then followed by detailed research of the heat treatment’s influences on the potency of the encapsulated form of Cholosens, i.e., the drug release rate, internalization of capsules by cells, and their dark and light toxicities.

## 2. Materials and Methods

### 2.1. Materials

Poly(sodium 4-styrenesulfonate) (PSS, M = 70 kDa), a 20% *w*/*w* water solution of poly(diallyldimethylammonium chloride) (PDADMAC, M = 100–200 kDa), dextran sulfate, sodium salt (DS, M > 40,000), poly-l-arginine hydrochloride (PArg, M > 70,000), α-chymotrypsin from bovine pancreas, calcium chloride dihydrate, anhydrous sodium carbonate, ethylenediaminetetraacetic acid trisodium salt (EDTA), rhodamine 6G (RhD6G), fluorescein 5-isothiocyanate (FITC), phosphate-buffered saline (PBS), Dulbecco’s minimum essential medium (DMEM), fetal bovine serum (FBS), Alamar blue, and calcein-AM were purchased from Sigma-Aldrich. Minimum essential medium (MEM), penicillin, streptomycin, trypsin, and trypan blue were purchased from Thermo Fisher Scientific. Hydrochloric acid was obtained from Merck. Zinc phthalocyanine (Cholosens) was kindly provided by the Institute of Organic Intermediates and Dyes (Moscow, Russia). All chemicals were used as received without further purification.

Normal human dermal fibroblasts (NHDF) and HeLa cell cultures were obtained from the Department of Cell Engineering, Education and Research Institute of Nanostructures and Biosystems, Saratov State University, Russia. *Staphylococcus aureus* and *Escherichia coli* were from ATCC (ATCC 25923 and ATCC 25922, respective strains).

Deionized water with specific resistivity higher than 18.2 MΩ cm^−1^ from a three-stage Milli-Q Plus 185 purification system was used in the experiments.

### 2.2. Preparation of Microcapsules and Loading with Cargo

A single batch of hollow PMC was prepared and used in all experiments described in this manuscript.

The capsules were assembled and loaded with Cholosens following the method previously described by our group [46]. In brief, the CaCO_3_ microparticle template was synthesized by mixing 2 mL of each of 1 M CaCl_2_ and Na_2_CO_3_ solutions under vigorous agitation for 30 s. The obtained CaCO_3_ spherical particles with (average diameter 4 μm) were collected by centrifugation and thoroughly washed with DI water. Multilayer capsules comprising four bi-layers of DS/PArg were then assembled on CaCO_3_ via the layer-by-layer (LbL) method. DS and PArg were alternatively adsorbed from 2 mg/mL and 1 mg/mL of respective aqueous solutions, also containing 0.5 M NaCl, starting from the DS layer. After each single layer formation, the particles were thoroughly washed with water to remove the uncoupled polymer. The obtained coated particles were treated with 5 mL of 0.2 M EDTA for 15 min to remove the inorganic phase resulting in the formation of hollow polymeric capsules. Capsule suspensions containing variable numbers of particles were then re-dispersed in 1 mL of an aqueous solution of Cholosens (0.05 mg/mL). After one hour of incubation needed for the infiltration of Cholosens, each suspension was divided into two specimens. Capsules in specimen 1 were immediately washed with DI water by centrifugation to remove the unloaded Cholosens, whereas capsules in specimen 2 were heated up to 80 °C and kept for 60 min at constant shaking (500 rpm) before cooling down for ten minutes and washing. The supernatants were collected to measure the concentration of Cholosens (the data were further used to calculate the encapsulation efficacy, and the amount of Cholosens loaded in PMC). Here and further in the manuscript, the concentration of Cholosens in supernatants was determined spectroscopically (Synergy H1 reader (BioTek, Winooski, Vermont, U.S.A.) by measuring the intensity of fluorescence at λ_ex_ = 685 nm, λ_em_ = 715 nm. The fluorescence intensity data were then converted to concentrations using a calibration curve plotted for a series of dilutions with a known concentration of Cholosens, which exhibited linear character in the measured concentration range. Each calibration solution was prepared in 1× PBS to match the ionic strength of the tested samples.

The PMC were post-loaded with RhD6G of FITC dyes via incubation of the microcapsule suspension in the respective solutions (0.1 mg/mL) for 60 min followed by three washing steps with DI water.

### 2.3. Capsule Enzymatic Degradation

Cholosens-loaded capsule suspensions were lyophilized in FreeZone 12 Labconco freeze drier. Each sample was then mixed with 1 mL of 1 mg/mL α-chymotrypsin dissolved in 1× PBS (pH 7.4) in 2 mL centrifuge tubes and kept at 37 °C for 24 h. Undissolved polymeric complexes were then extracted by centrifugation and resuspended again in the enzyme solution. The respective supernatants were analyzed with fluorescence spectroscopy to determine the amount of Cholosens released from the capsules after the complete digestion of the PMC.

### 2.4. Release of Cholosens

The cumulative release of Cholosens from 4.5 × 10^8^ [DS/PArg]_4_ microcapsules in 1 mL of 1× PBS (pH 7.4) was measured with fluorescence spectroscopy over 48 h. The medium was fully refreshed at each timepoint.

### 2.5. Uptake of Microcapsules by Cells

Uptake of PMC was studied on HeLa cells by flow cytometry. For this purpose, the capsules were labeled with FITC (λ_ex_ = 491 nm, λ_em_ = 516 nm). The cells were seeded in 24-well cell culture plates at the density of 10000/cm^2^ in 0.5 mL DMEM supplemented with 10% FBS and 1% penicillin–streptomycin appropriate for the cell passage and incubated for 24 h. The medium was then replaced by the fresh DMEM containing FITC-labelled PMC (5 capsules/cell). After incubation for 1, 2, 4, and 24 h to allow the capsule uptake, the cells were rinsed with PBS (pH 7.4) three times, trypsinized, centrifuged, and kept in ice-cold PBS until study with a FACSCalibur flow cytometer. The cells were treated with trypan blue to quench the extracellular fluorescence of FITC and to detect the signal from internalized capsules solely [56]. Quantitative data were obtained using the BD CellQuest software.

### 2.6. PDT Activity of Cholosens-Loaded Microcapsules on Human Cells

HeLa and NHDF cells were seeded in a 96-well cell culture plate at the density of 10^4^ cells per well. Each well in the culture plate was filled with 100 μL of MEM supplemented with 10% FBS, also containing 1% penicillin-streptomycin. The plates were incubated at 37 °C in 5% CO_2_ atmosphere. Twenty-four hours after plating, free or encapsulated forms of Cholosens were added to the culture medium followed by incubation overnight. The encapsulated form of Cholosens was represented by loaded [DS/PArg]_4_ shells with or without consequent heat exposure. PMC were added to the culture medium at the densities of 5, 20, or 40, and each capsule contained 0.9 ± 0.13 pg of Cholosens.

The cell culture medium was further replaced with PBS to reduce optical density. Each well of the plate was irradiated with a light-emitting diode of 680 nm wavelength (Polironik, Moscow, Russia) for 7.5 min with an intensity of 4000 lx (corresponding to 80 mW/cm^2^). Upon that, PBS was replaced by the cell culture medium again.

In the last step, 10 μL (10% *V*/*V*) of fluorescence dye Alamar blue was added to each well for detecting viable cells followed by 24 h of incubation. Fluorescence intensity of the samples (excitation 560 nm, emission 590 nm) was then measured spectroscopically.

Corresponding data on the viability of non-irradiated HeLa and NHDF cells grown without any of encapsulated or free forms of Cholosens were taken as 100%.

### 2.7. Antimicrobial PDT Activity of Photosensitizer-Loaded Microcapsules

The antibacterial activity of Cholosens-loaded PMC against the strains of *S. aureus* and *E. coli* was defined using the modified method of minimum inhibitory concentration. The experiments were conducted in triplicate to ensure statistical significance. Both *Staphylococcus aureus* and *Escherichia coli* were cultured and kindly provided by Saratov Institute of Traumatology and Orthopedics (Saratov, Russia). Nutrient agar was prepared and provided by the Institute of Organic Intermediates and Dyes (Moscow, Russia).

Next, 300 μL of suspensions containing microorganisms (3 × 10^5^ cells/mL) were mixed with microcapsule suspensions in different ratios (ranging from 3 to 80 capsules/cell) and incubated for 60 min. After that, the mixtures were irradiated with a 680 nm light-emitting diode, as described above. 100 μL of each light-treated suspension was then inoculated on the surface of NA obtained from 20 mL of 1.5% sterile NA medium solidified in the Petri dishes (2R = 7.5 cm). The Petri dishes were incubated for 24 h at 37 °C to grow individual bacterial colonies on the solid medium surface. The numbers of cells in the original suspensions were adjusted in such a way as to avoid confluence. Non-irradiated bacterial samples cultured in a similar way were used as a negative control. Corresponding data on the viability of non-irradiated *S. aureus* and *E. coli* grown without any of encapsulated or free forms of Cholosens was taken as 100%. The results were presented as arithmetic means and standard deviations.

### 2.8. Characterization

The obtained PMC were visualized with a scanning electron microscope (SEM): the FE SEM JSM6700F instrument in the secondary electron imaging mode at 5 keV. The samples were prepared by placing a 10 µL droplet of microcapsule suspension on a silicon substrate followed by drying overnight. The surface of the sample was then covered with a layer of gold before taking the SEM images.

The shell thickness was measured by atomic force microscopy (AFM) performed with an NT-MDT Ntegra spectra probe station in a semi-contact mode with the GOLDEN series probes NSG10 having a curvature radius of 6 nm. The samples for AFM measurements were prepared by drying 2 µL droplets of the capsule suspensions on the surface of a cover glass.

Capsule concentration in suspensions was obtained using a hemocytometer as an average of 5 measurements for each sample.

Capsule loading was measured via two complementary approaches. In the first approach, the supernatants collected after loading were analyzed spectroscopically. The cumulative loss of payload upon loading and consecutive washings was then deducted from its initial amount to obtain the weight of loaded Cholosens and loading efficacy. The loading efficacies of thermally treated vs. intact PMC were compared using the following equation:

$$E_{comp}\left(\%\right)=\frac{m{\left[\mathrm{Cholosens}\right]}_{heated}-m{\left[\mathrm{Cholosens}\right]}_{intact}}{m{\left[\mathrm{Cholosens}\right]}_{intact}}\times 100\%,$$

where $E_{comp}$—the comparative encapsulating efficacy of two approaches; $m{\left[Cholosens\right]}_{heated}$ and $m{\left[\mathrm{Cholosens}\right]}_{intact}$—the amount of Cholosens in heated and intact capsules, respectively.

In the second approach, the amount of Cholosens released from the enzymatically digested capsules was measured by fluorescence spectroscopy.

Complementary information on the capsule size and visual evidence of cellular uptake and cancer cell elimination by Cholosens-loaded PMC was provided by confocal laser scanning microscopy (CLCM) using Leica TCS SP8 X (Leica Microsystems, Wetzlar, Germany).

Before taking the images, each capsule sample was manually scanned along the z-axis in the fine-focus regime to reveal the focal plane where the shells displayed the darkest interior at the maximal outlined area. The focus was then set up on this plane, and the sample was imaged. Captured capsules displaying the darkest interior (at least 20 per each sample) were measured to reveal the average diameter.

HeLa cells were plated into a Petri dish and incubated for 24 h before staining with a calcein-AM dye (λ_ex_ = 495 nm, λ_em_ = 515 nm). For staining, 1 μm of calcein-AM solution with an initial concentration of 1 mg/mL was added per one milliliter of the culture medium to achieve 0.001 mg/mL of calcein-AM. The cells were then kept in an incubator for 30 min, followed by thorough washing by PBS to remove the excess of the dye. Upon that, the suspension of the Cholosens-loaded capsules (2 capsules/cell) or a Cholosens aqueous solution were added into the cell medium. After two hours of incubation, the CLSM images were taken. The amount of added free Cholosens was as such to level with the total amount of the photosensitizer in the capsules (assuming 0.9 pg of Cholosens loaded in a single capsule). The position on the z-axis for imaging of HeLa cells was found manually by scanning the observed group of cells in the fine-focus regime to reveal the ventral area of the cell, which typically had the strongest signal from the calcein-AM dye (green fluorescence) and the largest observed area with the green fluorescence. This technique ensured that the observed Cholosens-loaded PMC (red fluorescence) were internalized by cells without the necessity of co-localization of the capsules with the cell nucleus. Cholosens-loaded [DS/PArg]_4_ microcapsules were visualized at λ_ex_ = 685 nm, λ_em_ = 715–780 nm. Photodynamically-induced damage of HeLa cells by different forms of Cholosens was visualized by irradiation of the respective samples with white laser (WLL E, avg. power 1.0 mW) at 670 nm. A single cell in the treated area received approximately 0.1 mW irradiation dose.

### 2.9. Data Analysis

Statistical analysis of data was conducted using unpaired two-tailed Student’s t-test. Data are presented as the means ± standard deviations (SD). Two levels of significance were established (*p* < 0.05 and < 0.01).

## 3. Results

### 3.1. Temperature-Induced Morphological Changes in the [DS/PAgr]_4_ PMC System

**Figure 2:**
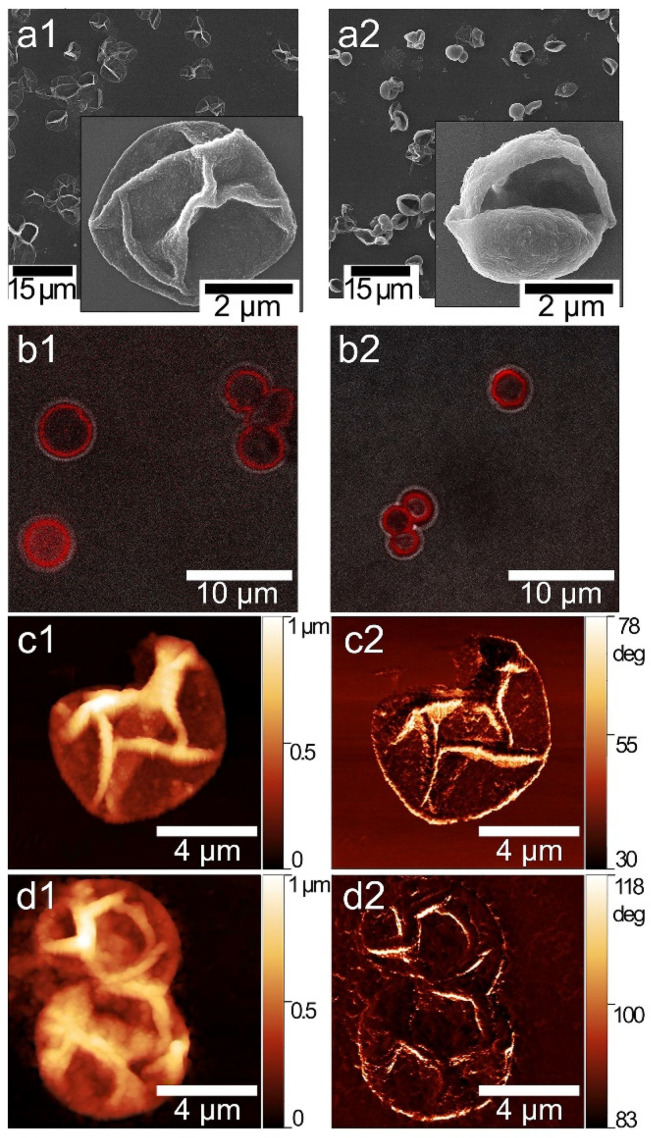
(**a1**,**a2**) SEM and (**b1**,**b2**) CLSM images of [DS/PArg]_4_ microcapsules: (**a1**,**b1**) intact; (**a2**,**b2**) after heat treatment at 80 °C for 60 min. The capsules were post-loaded with RhD6G for visualization with CLSM at λ_ex_ = 525 nm, λ_em_ = 548 nm. (**c1**,**d1**) and (**c2**,**d2**) AFM images and respective phase contrast of the capsules (**c1**,**c2**) before and (**d1**,**d2**) after heat treatment.

We have previously determined a temperature treatment profile leading to size reduction and tightening of PMC capsules at 90 °C for 60 min [36]. In this case, to ensure the stability and biological efficacy of Cholosens, we lowered the temperature to 80 °C while keeping the treatment duration unchanged. Images obtained by scanning electron microscopy (SEM), confocal laser scanning microscopy (CLSM), and atomic force microscopy (AFM) in Figure 2 depict the [DS/PArg]_4_ microcapsules before and after heating to validate and quantify the PMC response to treatment at the modified conditions. Upon exposure to 80 °C for 60 min, the capsules underwent morphological changes that we observed previously, i.e., a decrease in size and tightening [36]. SEM images (Figure 2a1,a2) reveal the overall size reduction and (significantly) increased thickness of the heat-treated polymeric multilayer shells compared to intact PMC. Capsules also gained rigidity after heat exposure so that the smallest particles in the batch were able to stay spherical after drying on a solid support. In addition, SEM confirms the transformation of all capsules in the batch in response to 80 °C.

Estimations of the average capsule size using the CLSM images (Figure 2b1,b2) and respective cross-sections (for example see Figure S1) revealed that the capsules contracted after heating from 4.95 ± 0.65 μm to 3.78 ± 0.4 μm; that gives the size decrease of ~23%. In this study, the heated shells demonstrated an increase in thickness from 0.073 ± 0.009 μm to 0.114 ± 0.025 μm (~35% increase) according to the AFM data (Figure 2c1,c2,d1,d2).

### 3.2. PMC Loading and Release of Cholosens

Suspension of loaded capsules gained a bright blue color after the diffusion of Cholosens. Free Cholosens has two absorbance peaks at 635 and 680 nm. Figure S2 depicts the absorbance spectrum of Cholosens after the encapsulation. The positions of absorbance peaks of the encapsulated drug did not significantly shift when compared to those of the free drug, indicating that no structural change occurred upon the loading process.

Cholosens loading was assessed by the quantitative analysis of the loading efficiency that was essential for administration dosage of heated and intact encapsulated drug forms for in vitro studies. Previously we showed that the amount of rhodamine B detected in DS/PArg capsules was lower for heated analogs in comparison with the untreated PMC. Moreover, the loading of heated capsules did not depend much on the capsule concentration that was varied in the range of 6.9 × 10^7^–3.4 × 10^9^ capsules/mL, whereas the loading of non-treated PMC gradually increased with the increasing of the number of capsules in the sample [46]. The particular aspects of loading Cholosens in [DS/PArg]_4_ PMC were discovered upon varying the capsule concentration from 0.6 × 10^8^ to 9 × 10^8^ capsule/mL at a constant concentration of Cholosens (0.05 mg/mL).

**Figure 3:**
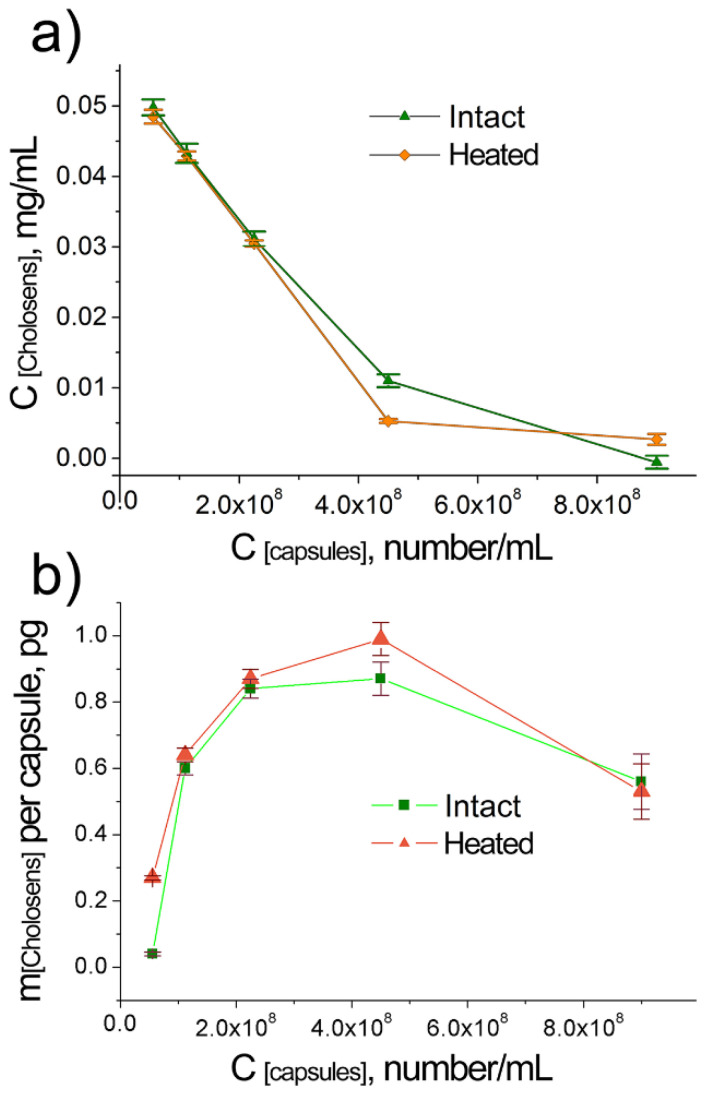
Loading of Cholosens in [DS/PArg]_4_ microcapsules with and without heat treatment (80 °C, 60 min) depending on the capsule concentration: (**a**) spectroscopically measured concentration of Cholosens remaining in supernatant after loading; (**b**) calculated amount of Cholosens in pg loaded in a single capsule.

The amount of Cholosens loaded in the [DS/PArg]_4_ microcapsules was measured by two complementary approaches, i.e., (1) by measuring the residual concentrations of the drug in the supernatants obtained after loading (Figure 3), and (2) by measuring the concentrations of the drug released after enzymatic degradation of the loaded PMC (Figure S3). Spectroscopic measurements of the supernatants obtained after the loading of Cholosens (Figure 3a) revealed that the residual concentration of the drug had decreased with increasing the capsule concentration for both heated and intact PMC. The minimal concentrations of Cholosens were detected in the samples containing the highest amounts of intact or heated PMC, i.e., 9 × 10^8^ capsules/mL. At this capsule concentration, the residual concentrations of Cholosens were 2.2% and 6.2% of the initial 0.05 mg/ml for intact and heated capsules, respectively.

The loading data obtained through measurements of Cholocesns released from enzymatically-degraded capsules (Figure S3a) and through the analysis of its residual amount in the supernatant (Figure 3a) are generally in good agreement and so is the respective data on the loading of a single capsule (Figure 3b and Figure S3b) calculated from the corresponding datasets plotted in Figure 3a and Figure S3a. In essence, capsules in bigger batches do entrap larger amounts of Cholosens. A sharp decrease in the loading of a single capsule, however, was observed at the biggest concentration of 9 × 10^8^ capsules/mL. The observed result contains no contradiction if we take into account the residual concentrations of Cholosens observed for the capsule concentration 4.5 × 10^8^ capsules/mL, which were approximately 20% and 10% of the initial amount in suspension for intact and heated capsules, respectively. As such, at a higher capsule concentration, the drop in the loading per capsule was well-expected because almost the same amount of Cholosens was spread across a larger number of capsules.

**Figure 4:**
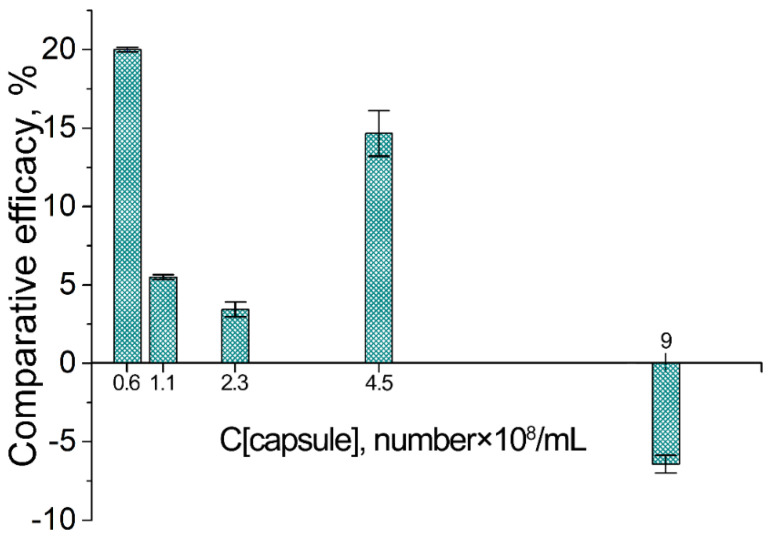
Loading of Cholosens in [DS/PArg]_4_ microcapsules with and without heat treatment (80 °C, 60 min) depending on the capsule concentration: comparative loading efficacy for heated vs. intact capsules. The loading efficacies of heated vs. intact PMC were compared using the data on the Cholosens loading in a single capsule displayed in Figure 3b.

The total amount of loaded Cholosens significantly increased when increasing the capsule concentration for both intact and heated capsules (Figure 3a, Figure S3a). Besides, the amounts of loaded Cholosens in the DS/PArg heated and intact microcapsules were close or slightly higher in the case of heated PMC for the majority of the capsule concentrations (Figure 4). At 9 × 10^8^ capsules/mL, intact PMC displayed better loading than their heated counterparts.

**Figure 5:**
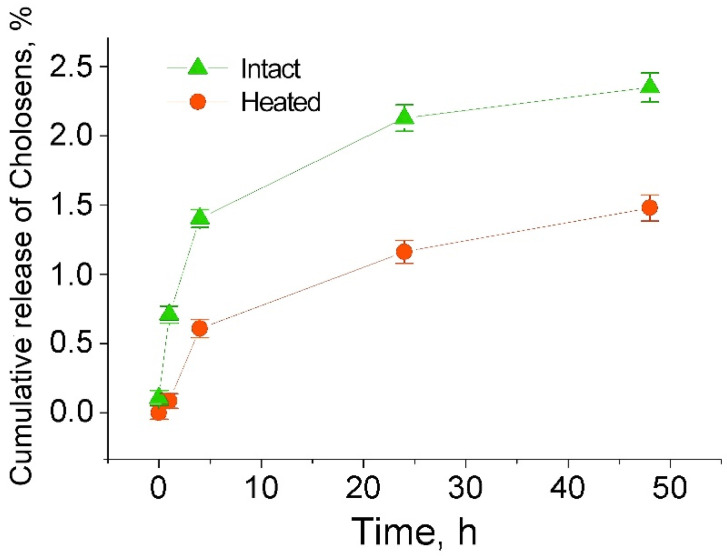
Cumulative release of Cholosens from 4.5 × 10^8^ [DS/PArg]_4_ microcapsules intact or treated by elevated temperature upon loading (80 °C, 60 min) in 1 mL of 1 × PBS buffer.

Figure 5 shows the respective release profiles of Cholosens from intact and heated capsules over 48 h. The amount of Cholosens released from the intact PMC at each timepoint was roughly about two times higher than from the heated ones, thereby emphasizing the effect of polymeric multilayer network tightening for the diffusion of Cholosens upon exposure to 80 °C.

### 3.3. Cellular Uptake of [DS/PArg]_4_ Microcapsules

**Figure 6:**
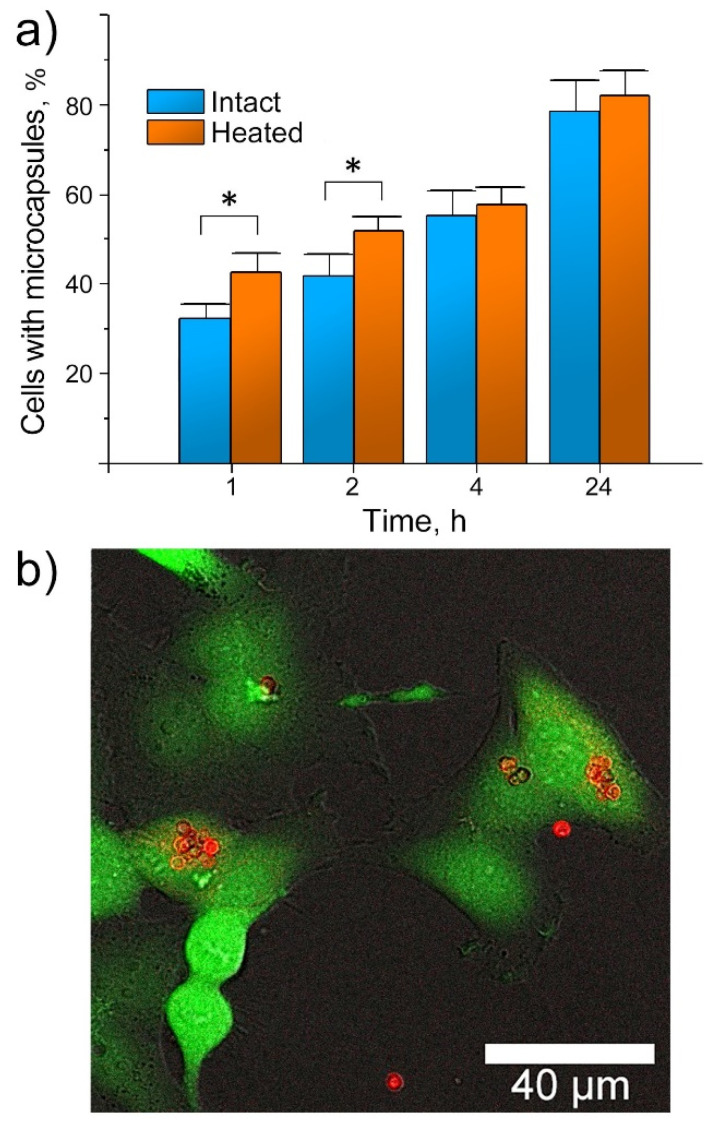
Interactions of [DS/PArg]_4_ microcapsules with HeLa cells: (**a**) flow cytometry data on the capsule internalization (10 capsules/cell) showing percentages of cells with at least one capsule (* *p* < 0.05, intact vs. heated capsules with the same duration of incubation); (**b**) characteristic CLSM image of HeLa cells incubated with heat-treated [DS/PArg]_4_ microcapsules loaded with Cholosens (three capsules/cell, 24 h, 37 °C). Green color displays the calcein-AM dye metabolized by viable cells (λ_ex_ = 495 nm, λ_em_ = 515 nm), and red color displays Cholosens encapsulated in [DS/PArg]_4_ microcapsules (λ_ex_ = 685 nm, λ_em_ = 715–780 nm).

Before engaging in the in vitro experiments, we studied the morphology of [DS/PArg]_4_ microcapsules in the cell culture medium. As such, we observed no changes for the capsules suspended in water or 1× phosphate-buffered saline (PBS). Capsule cellular internalization was then studied on HeLa cells by analyzing approximately 10^4^ cells per sample by flow cytometry. The PMC were post-loaded with FITC for analytical purposes. In each sample, the cells were mixed with capsules in a concentration of 10 capsules/cell. The uptake level (the proportion of cells (%) with at least one capsule) was determined as the median fluorescence intensity in each sample related to the median fluorescence intensity of the control cells incubated without capsules. The results are reflected in Figure 6a as mean values ± standard deviations.

Internalization of [DS/PArg]_4_ microcapsules by HeLa cells was a time-dependent process, wherein the number of cells with internalized capsules had gradually increased. Similarly to the data described elsewhere [57], the highest capsule internalization rate by HeLa cells was observed during the first four hours (Figure 6a). The uptake efficacy was slightly higher for heated capsules than for intact ones over the early two hours (* *p* < 0.05); no significant difference in the uptake of heated and intact PMC was observed at the next timepoints, indicating no prolific impact of the capsule size difference (Table S1). After 24 h, nearly 82% of the studied HeLa cells had internalized the [DS/PArg]_4_ microcapsules. The emission spectrum of Cholosens has a sharp peak at ~700 nm wavelength that allows visualization of the Cholosens-loaded PMC in the cell culture medium and inside the cells after internalization. A typical CLSM image of the cells containing Cholosens-loaded capsules at 24 h timepoint supports the flow cytometry data (Figure 6b).

### 3.4. Comparison of the Actions of the Encapsulated and Free Forms of Cholosens on Human Cell Lines

The cytotoxicity of the free and the [DS/PArg]_4_-encapsulated Cholosens was studied on the normal (NHDF) and cancerous (HeLa) human cell lines by varying the capsule/cell ratio from 1 capsule/cell up to 40 capsules/cell. HeLa and NHDF cells were cultured overnight in the Cholosens containing medium or with the Cholosens-loaded PMC; then, the number of viable cells was measured by detection of the metabolic activity through staining the cells with a resazurin-based dye, Alamar Blue. Control groups for both cell lines were incubated in the culture medium with no added capsules or free drug at 37 °C. After 24 h, the medium was exchanged for 1× PBS buffer before cell irradiation with a laser diode to avoid absorption of light by the medium.

**Figure 7:**
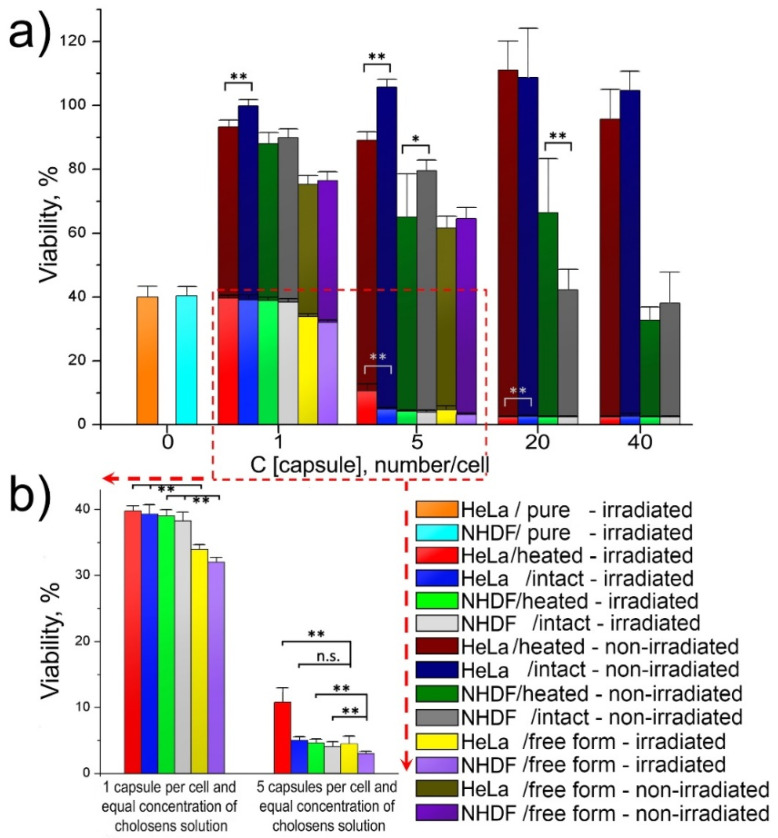
Light and dark viability of HeLa and NHDF cells in the presence of free and encapsulated forms of Cholosens: (**a**) cell viability depending on the number of Cholosens-loaded [DS/PArg]_4_ microcapsules (intact or heated) in the culture medium; (**b**) comparative effect of the free and encapsulated forms of Cholosens after light irradiation. HeLa/pure and NHDF/pure refer to the cells untreated with any form of Cholosens. Corresponding data on the viability of non-irradiated HeLa and NHDF cells grown without any of encapsulated or free forms of Cholosens were taken as 100%. The respective Student’s t-test results are shown in Tables S2–S6. * *p* < 0.05, ** *p* < 0.01 when compared heated vs. intact capsules (**a**), and encapsulated forms of Cholosens vs. free drug (**b**). The respective IC50 values for each drug form are shown in Table S10.

Non-encapsulated Cholosens possessed concentration-dependent toxicity for both studied cell lines, which was markedly reduced after the drug encapsulation in [DS/PArg]_4_ (Figure 7a). Both intact and heated encapsulated forms of Cholosens did not affect the viability of HeLa cells at all studied capsule/cell ratios. As suggested by Figure 6b, HeLa cells internalized a large number of capsules with no adverse effects on morphology. The captured cells display a well-defined cell shape spread typical for attached HeLa cells. After 24 h of incubation with PMC, the cells exhibit sufficient adhesion, indicating that they were healthy and that they tolerated the internalized capsules well. The NHDF cells became significantly inhibited by the encapsulated forms of Cholosens at the capsule concentration of 20 capsules/cell (Figure 7a). At one and five added capsules per cell, the Cholosens-loaded capsules were significantly less cytotoxic than the non-encapsulated drug (Table S2).

Light cytotoxicity of the encapsulated and free forms of Cholosens was measured comparatively after irradiation of the respective wells with adherent HeLa and NHDF cells with a 680 nm light-emitting diode for 7.5 min at the power density of 80 mW/cm^2^ over the area of ~0.8 cm^2^. The data in Figure 7a demonstrate a significant decrease in fluorescence signal (i.e., the metabolic activity) in the control groups for both HeLa and NHDF cells in response to the light irradiation: the residual viability of the treated cells was ~40%. Cell treatment with encapsulated and free Cholosens (equal to five capsules/cell and higher) resulted in a drastic drop in the number of living cells of both cell lines after light irradiation when compared to the non-irradiated cells.

The effects of the encapsulated Cholosens in both heated and intact [DS/PArg]_4_ PMC compared to the free drug form on the cell viability varied by up to 6–7%, except in the case of HeLa cells treated with five intact capsules per cell; the viability of those was significantly different from the treatment with free Cholosens (Figure 7b). It appears that at this capsule/cell ratio, the amount of Cholosens delivered by capsules is not sufficient to provide the desired effect, but it is working as projected at higher ratios. At the lowest capsule/cell ratio (i.e., 1 capsule/cell), the free form of the drug also exhibited a limited advantage over the encapsulated form (Figure 7b, Table S2). The results of the Student’s t-test showed that laser irradiation provided a significantly higher level of toxicity of the free drug form in both cell lines (Table S3). Additionally, a high level of statistical significance (** *p* < 0.01) of the difference between the toxicity of intact capsules after laser irradiation compared to non-irradiated samples was confirmed for all studied capsule/cell ratios in both cell lines (Table S4). In the majority of cases examined, the toxicity of the intact and heated Cholosens-loaded capsules is not significant (Table S5). This phenomenon was observed both for the non-irradiated and irradiated conditions in HeLa and NHDF cells. Rare cases of significant differences between intact and heated capsules are marked in Figure 7a.

Cells were monitored by CLSM to detect photodynamically-induced damage of HeLa cells by different forms of Cholosens. First, the cells were incubated in the Cholosens solution for a few hours, followed by staining with a vital dye, calcein-AM. The cells were then irradiated at 670 nm for 60 s over the sample area of ~175 × 175 μm^2^ to induce the generation of singlet oxygen by the photosensitizer. HeLa cells incubated in the culture medium without adding Cholosens for the same duration were used as a negative control after replacing the medium with 1× PBS buffer before light irradiation.

**Figure 8:**
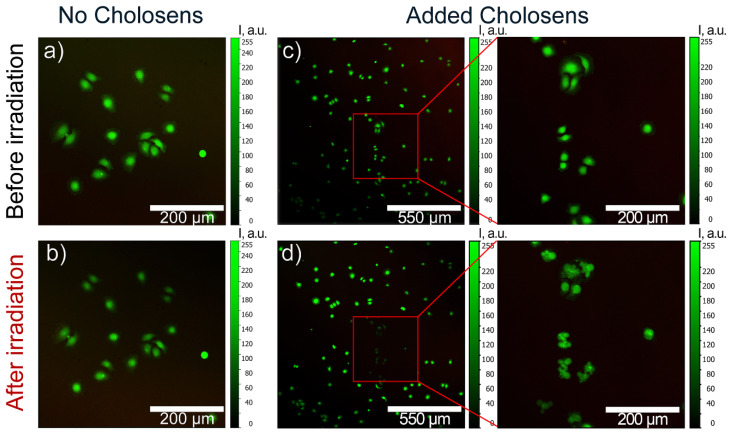
CLSM images of calcein-AM-stained HeLa cells showing the photodynamic effect of a free form of Cholosens added an amount equal to that of the encapsulated drug at the concentration of three capsules per cell: (**a**,**b**) cells with no added Cholosens before and after light irradiation at 670 nm for 60 s, respectively; (**c**,**d**) cells in the Cholosens solution before and after light irradiation at 670 nm for 60 s, respectively. Red squares in the images (**c**,**d**) outline the irradiated area in the sample containing Cholosens. Zoom-in images of the outlined area before and after light exposure are shown to the right of the individual images (**c**,**d**). Color scale from 0 to 255 a.u. depicts the fluorescence intensity for a direct comparison of the images.

The control sample did not display any visible changes in the cell morphology after the irradiation (Figure 8a,b). In the presence of Cholosens, however, the irradiated cells underwent dramatic morphological changes even five minutes past the beginning of the light exposure, which was accompanied by the formation of the vesicles over their surfaces (Figure 8c–f). Distinct transformations of the cell shape and a consequent decrease in the fluorescence of calcein-AM over the next 20 min indicate the destruction of the cells within the treated area, confirming the high potency of Cholosens as a PDT drug (Figure 8c–f).

**Figure 9:**
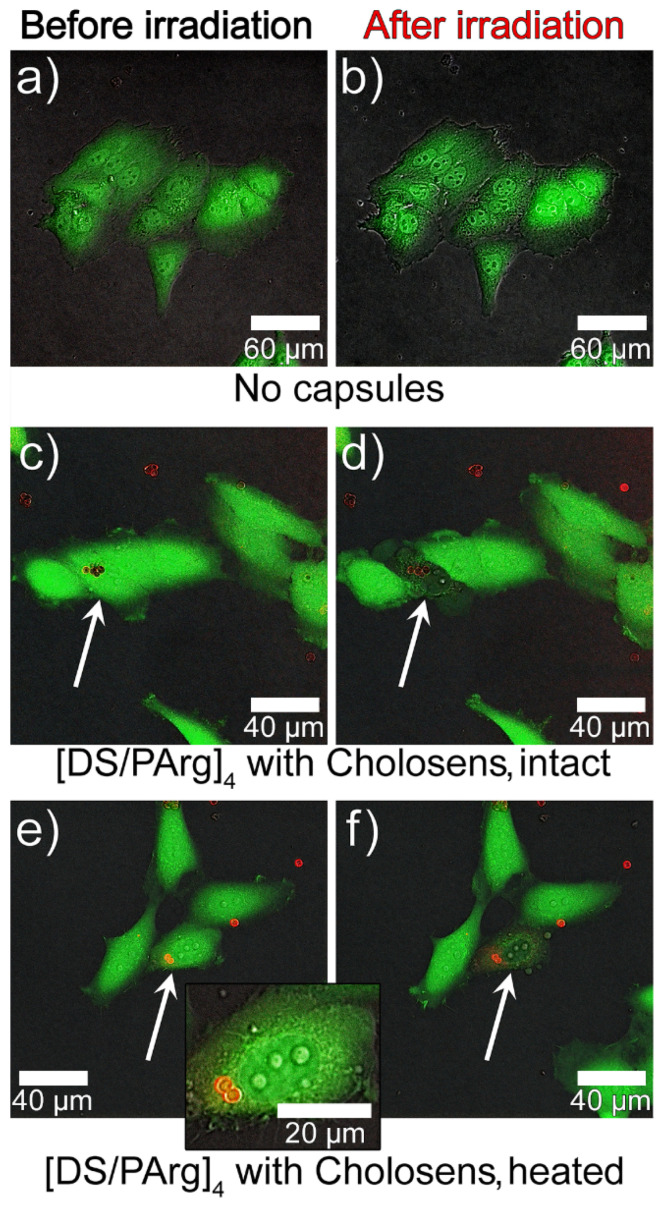
CLSM images of calcein-AM-stained HeLa cells (green fluorescence) showing the photodynamic effect of the encapsulated forms of Cholosens (red fluorescence): (**a**,**b**) control cells incubated without PMC; (**c**–**f**) cells incubated with [DS/PArg]_4_ microcapsules ((**c**,**d**) intact, (**e**,**f**) heated) added at the concentration of three capsules/cell (37 °C, 24 h) before and after light irradiation at 670 nm for 60 s, respectively. White arrows point to the individual cells, which were exposed to light irradiation. The capsules were added in the amount of three capsules/cell.

The light-induced cell elimination process by intact and heated encapsulated forms of Cholosens was visualized using the same negative control as in the previous experiment, i.e., HeLa cells first cultured in the medium with no added encapsulated or free photosensitizer and then transferred to 1× PBS buffer right before the light irradiation. The control cells irradiated for 60 s with the 670 nm white laser displayed no destructive processes 20 min after the light was switched off (Figure 9a,b).

Three capsules per cell was chosen as an optimal concentration for the CLSM experiments based on the convenience of focusing on a single cell with internalized capsules. It would be harder to accomplish this precision at higher capsule concentrations when the sample was saturated with such cells. In addition, as suggested by Figure 7a, at three capsules per cell, the viability of non-irradiated cells was not affected. For visualization of the encapsulated forms of Cholosens, we picked up the area of 100 × 100 μm^2^ that included the cells with and without internalized capsules. Similarly to the control sample, the cells in the selected area were irradiated for 60 s, followed by observations and imaging 20 min after the light treatment was over (Figure 9c–f). The corresponding images in Figure 9 depicting HeLa cells with internalized intact or heated PMC show a distinct decrease in the fluorescence intensity of calcein-AM, which became apparent already after the first 30 s from the beginning of light treatment. Light irradiation caused the formation of vesicles in some of the treated cells with encapsulated Cholosens, which may be a sign of apoptosis (Figure 9f). Besides, the images c–f in Figure 9 also suggest that even a couple of the drug-loaded capsules inside the cell was enough to cause light-induced cell destruction.

As suggested by Figure 7, both HeLa and NHDF cells cultured with no added encapsulated or free Cholosens were affected by laser treatment, so one might expect to see morphological changes in HeLa cells under CLSM (Figure 8a,b and Figure 9a,b). It must be mentioned for this reason that the cells irradiated with the CLMS laser (Figure 8, Figure 9) received considerably lower irradiation dosages compared to those treated with the light-emitting diode (Figure 7) due to a shorter time of exposure and a lower irradiation power, which was below the threshold to overcome for morphological damage.

### 3.5. Effect of the Encapsulated Forms of Cholosens on Bacterial Cells

**Figure 10:**
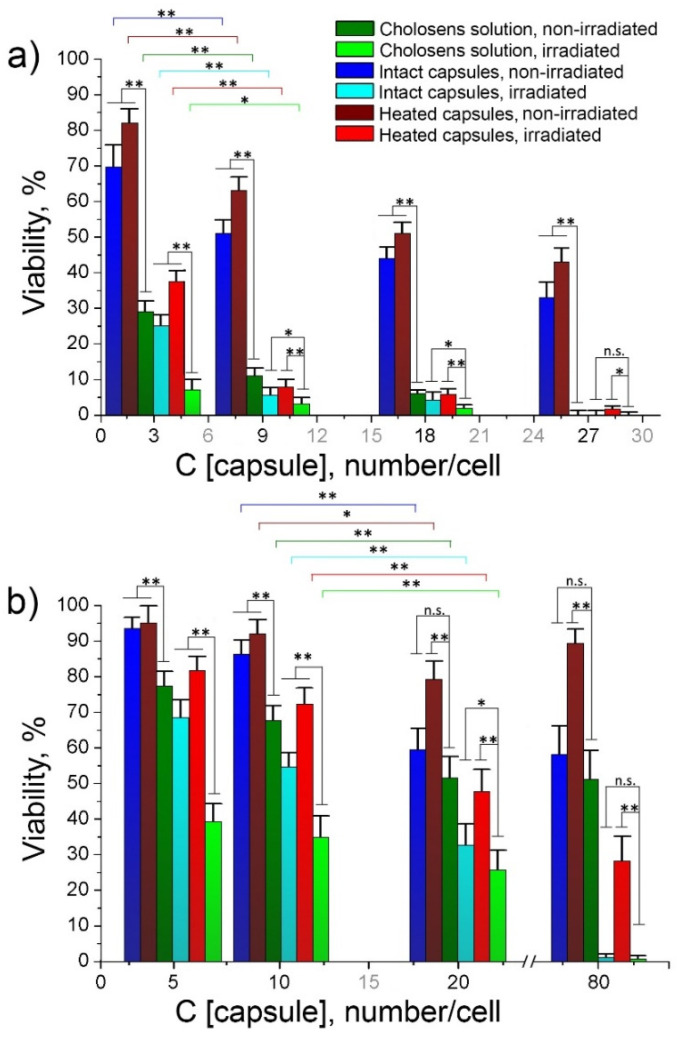
Light and dark viability of bacterial cells depending on the number of Cholosens-loaded capsules (intact or heated) added to the culture medium: (**a**) Gram-positive *S. aureus*; (**b**) Gram-negative *E. coli*. Corresponding data on the viability of non-irradiated *S. aureus* and *E. coli* grown without any of encapsulated or free forms of Cholosens were taken as 100%. The figure legend is uniform across the graphs **a** and **b**. The statistical significance of the differences between the observed results was determined against the corresponding Cholosens solution with or without laser irradiation and between the samples containing different numbers of capsules: three and nine capsules/cell (**a**) and 10 and 20 capsules/cell (**b**) (*n* = 6, * *p* < 0.05, ** *p* < 0.01). The respective Student’s t-test results are shown in Tables S7–S9. The respective IC50 values for each drug form are shown in Table S11.

As claimed by the manufacturer, Cholosens is a potent antimicrobial PDT drug against *Helicobacter pylori*, *Campylobacter jejuni*, *Escherichia coli 1257*, *Escherichia coli 675*, *Enterococcus faecalis*, *Staphylococcuc aureus*, and some other pathogens. Here we compared the efficacies of free and encapsulated Colosens to inhibit a Gram-positive bacterial strain of *S. aureus* (Figure 10a) and a Gram-negative bacterial strain of *E. coli* (Figure 10b). The bacterial cells were incubated in a Cholosens solution or the drug-loaded capsule suspensions for one hour before light irradiation, and viability measurements against the untreated microorganisms kept without Cholosens. The same samples were also studied without light irradiation to evaluate the dark toxicity of the respective drug forms.

In our study, free Cholosens in solution revealed significant dark toxicity to *S. aureus*. The viability of bacterial cells incubated with Cholosens dropped below 30% even at the lowest concentration studied, which was equal to the amount of drug provided by three capsules per cell. The highest studied concentration of Cholosens thus was equivalent to the amount of the drug loaded in 27 capsules per cell, at which the cell viability dropped to almost 0% (Figure 10a). Predictably, *E. coli* showed higher resistance to Cholosens conferred by the cell wall. The viability of *E. coli* cells was between 75% and 50%, depending on the drug concentration that was ranging from five to 80 capsules per cell (Figure 10b).

The dark toxicity of both encapsulated drug forms to *S. aureus* decreased gradually from ~80% to ~35% in the investigated capsule density range, which was significantly lower than the dark toxicity of free Cholosens. Besides, intact PMC possessed a significantly higher inhibition efficacy than the heated ones (Table S7). Both encapsulated forms revealed the antimicrobial activity after light irradiation, which is approaching the light toxicity of free Cholosens at the concentration equal to nine capsules per cell or higher. In this concentration range, the efficacies of heated and intact PMC were level as per the Student’s t-test (Table S7).

For *E. coli,* the dark toxicity of heated [DS/PArg]_4_ microcapsules against this bacterial strain was around 90% (of viable cells with respect to control) and lower compared to the dark toxicity of intact PMC over the whole studied density range. On the contrary, intact capsules gradually became more toxic upon increasing the capsule-to-cell ratio. Maximal light toxicity for *E. coli* achieved with heated PMC was the ~28% of cells remained viable. The light-induced toxicity of intact capsules was significantly higher than of their heated counterparts for all capsule/cell ratios (Table S8). The same trend and level of significance were observed for non-irradiated samples with the only exception at five capsules/cell (Table S8). An increase in the capsule/cell ratio from three to nine leads to a significant decrease in the viability of Gram-positive *S. aureus* microorganisms; as for Gram-negative *E. coli*., a significant decrease in viability was achieved by increasing the capsule/cell ratio from 10 to 20 (Table S9). These differences between the respective datasets are reflected in Figure 10. Thus, the intact encapsulated form of Cholosens nearly matched the free drug inhibitory effect on *E. coli* at the density of 20 capsules per cell, eliminating almost 70% of microorganisms; it further induced almost full elimination of the bacteria at 80 capsules per cell.

## 4. Discussion

This study has developed biodegradable [DS/PArg]_4_ microcapsules assembled on the sacrificial CaCO_3_ vaterite template as a delivery platform for Cholosens, a novel PDT drug. Cholosens was loaded on PMC via a classic post-loading method through the diffusion of drug molecules into the capsules resuspended in the Cholosens solution. Loaded capsules were further heated at 80 °C for 60 min to study the effect of the heat treatment on the loading efficacy and photodynamic properties of the encapsulated form of Cholosens.

Our results suggest that the chosen temperature regime leads to the morphological transformation of capsules upon heat exposure, i.e., tightening of the polymeric multilayer network and capsule contraction. It has to be noted that such treatment does not cause any adverse effects on the optical and photodynamic properties of Cholosens.

In contrast to a poor loading of rhodamine B in DS/PArg microcapsules upon heating [46], Cholosens was loaded to the PMC with high efficacy, when the total loading gradually enhanced upon capsule concentration increase. For the highest concentration studied here (9 × 10^8^ capsules/mL) the loading efficacy exceeded 90%. Besides, unlike what was observed for rhodamine B, the loading per capsule was higher for heated capsules compared to intact ones all the way until the capsule concentration reached 9 × 10^8^ capsules/mL (Figure 4). At this number of capsules, the loading efficacy of heated vs. intact capsules showed an opposite trend. Similarly to how it was previously explained for the loading of rhodamine B [46], the total amount of the Cholosens molecules displaced from the shell into supernatant upon the capsule heating increased with the increase of the capsule concentration, causing an overall comparative decrease of encapsulation efficacy in respect to intact PMC. Eventually, at some point (9 × 10^8^ capsules/mL in case of Cholosens), the number of displaced dye molecules will overcome the amount of those entrapped in the capsules. The data in Figure 3 and Figure 4 are likely to feature a much stronger affinity of Cholosens in comparison to rhodamine B to the outer charge compensation within the polymeric multilayer network. A possible reason here is a high affinity of multiple Cholosens ionic centers to the counterions of the polymeric network in the capsule wall. Unlike rhodamine B, which contains only a single ionic pair in the neutral state, Cholosens has seven or eight of them, which essentially makes each molecule a potential cross-linker, thereby greatly improving the rigidity of the PMC wall and its resistance toward contraction upon heat application.

While the heat treatment did not affect the loading, it had an impact on the release of Cholosens from PMC. The heated capsules released the drug slower than their intact counterparts, which was also a reason for their lower antimicrobial PDT activity in comparison to the untreated PMC (c.f. discussion later in the text).

The developed Cholosens-loaded [DS/PArg]_4_ microcapsules and free drug were comparatively studied on eucaryotic cells (human normal and cancerous cells, NHDF and HeLa, respectively) and bacteria (*S. aureus* and *E. coli*); we aimed to determine the cytotoxicities, bacterial toxicities, and anticancer and antimicrobial PDT efficacies of different drug forms.

Non-encapsulated Cholosens inhibited the metabolic activity of both human cell lines, which we attributed to its electrostatic binding to the plasma cell membrane. Upon loading of Cholosens in [DS/PArg]_4_ microcapsules, the drug toxicity was significantly mitigated, thereby providing distinctive evidence of the postulated need for Cholosens encapsulation.

We figured out that capsules mainly deliver the photosensitizer to NHDF and HeLa cells through cellular uptake. The internalization of capsules 4–6 µm in diameter by cancerous cells, including HeLa, has been well-described in literature [57,58], supporting our flow cytometry data (Figure 6a). Both intact and heated PMC loaded with Cholosens displayed no dark toxicity to HeLa cells (Figure 7, Table S6), which internalized a significant number of capsules while remaining healthy and attached to the surface of the culture plate. Upon the light irradiation, both encapsulated drug forms showed strong PDT activity in HeLa and NHDF cells, starting from five capsules per cell. Although differences between the efficiencies of encapsulated and free Cholosens upon the light irradiation are statistically significant for both cell lines (Table S3), the viability is negligible for all samples (for five capsules per cell the viability varies within 3–5%, except for the 10% observed for Cholosens-loaded heated capsules in the Hela cell line). We also observed that just a few internalized capsules were enough to eradicate the HeLa cell after light irradiation.

NHDF cells poorly tolerated the encapsulated drug forms, showing viability below 80% already at five added capsules per cell. This result indicates the necessity for a more profound understanding of the mechanism of the capsule-cell interactions through in vitro and in vivo studies, while it highlights the importance of cancer-targeting by delivery systems. Besides, further research on the design of PMC for delivery of Cholosens or analogs needs to consider a short lifetime of singlet oxygen (about a few µs or less), so its oxidative effect cannot be extended past a proximate locale. Thus, the cell could either undergo apoptosis or necrosis depending on which crucial organelle or functional molecule (e.g., proteins, DNA, etc.) was affected by the delivered drug. For example, damage in DNA [59,60,61] or the mitochondrial membrane [62,63] leads to apoptosis, while defects induced in the cytoplasmic membrane can trigger the necrotic path of cell inactivation [64]. A deeper understanding of the capsule fate after cell internalization would also help to optimize the design, and thus, the efficacy of PMC as a carrier-system for anticancer PDT drugs.

As for the antimicrobial activity, free Cholosens inhibited the growth of *S. aureus* and *E. coli* without activation by light significantly higher than both encapsulated forms at most of the studied capsule densities and drug concentrations. (Tables S7 and S8). By virtue of its cationic nature, Cholosens inhibits microorganisms via electrostatic binding to negatively-charged bacterial membranes. The [DS/PArg]_4_ composition is supposed to have a positively-charged surface due to the outmost layer made of PArg. Our findings, however, reveal that such capsules often display negative zeta-potential, with its absolute value increasing after thermal treatment. This phenomenon warrants a separate study beyond the scope of the current manuscript. We wanted to mention this aspect only because it one of the likely reasons for the observed difference in dark antimicrobial efficacy that was established for free and encapsulated forms of Cholosens. Besides, the electrostatic interactions between the positively charged capsule surface and the negatively charged bacterial membrane are compromised by the presence of a large number of solvated counterions at the surfaces, which could potentially deter the electrostatic interactions of the large ionic species. Thus, the capsule size, geometry, and surface charge density will play a crucial role in the reported here lesser efficacy of Cholosens-loaded PMC vs. the drug itself.

Unlike the cancer cells, bacteria are unable to uptake capsules due to their small size (about a few microns). The current mechanism of action for many antibacterial drugs includes an initial attachment of the oppositely-charged ionic drug to the bacterial surface by electrostatic interaction. Aside from the size disparity, PMC-loaded Cholosens has a much more complex and better-balanced ionic interaction of Cholosens molecules and polymeric layers in the network and/or inside the capsules than the pure drug. Therefore, not only the size of the PMC-loaded Cholosens but the reduced ionic interaction of the loaded capsules with the bacterial surface makes the pure drug more prone to get attached to the bacteria, further providing a desired photodynamic efficacy. The results of our study are entirely consistent with the proposed mechanism of antimicrobial activity of the developed capsules. Both heated and intact capsules loaded with Cholosens displayed the antimicrobial PDT activity upon irradiation with 680 nm light against *S. aureus* and *E. coli*, which increased with increasing the capsule/cell ratio. At most studied capsule densities, intact PMC had a higher inhibition activity than the heated ones, which was likely due to an approximately two-times faster release of Cholosens from the intact capsules compared to their heated counterparts. Lower inhibition level for *E. coli* in comparison with *S. aureus* displayed by the free and encapsulated Cholosens is predictable and consistent with the presence of cell walls in the Gram-negative microorganisms responsible for higher resistance of such bacteria to chemical treatment. Besides, we demonstrated that at specific capsule densities, their antimicrobial PDT activity matches the antimicrobial activity of the free drug.

Our study introduces the DS/PArg PMC as an efficient delivery system for the water-soluble PDT drugs. Further development of the encapsulated drug forms would be aimed at the tailoring mechanisms for controlled delivery, e.g., capsule size reduction for efficient tumor targeting via the EPR effect. We believe that PMC produced via consecutive surface self-assembly of complementary biocompatible, naturally-derived polyions will outperform the vast majority of nanomaterials in the treatment of infectious diseases and cancer due to the stable structure and favorable safety profiles.

## Supplementary Materials

The following are available online at https://www.mdpi.com/1999-4923/12/7/610/s1. Figure S1: Fluorescence intensity cross-section profiles of the intact and heated capsules labeled with RhD6G (λ_ex_ = 525 nm, λ_em_ = 548 nm). Figure S2: The absorbance spectrum of Cholosens. Figure S3: Loading of Cholosens in [DS/PArg]_4_ microcapsules with and without heat treatment (80 °C, 60 min) depending on the capsule concentration. Tables S1–S9: Student’s t-test results for Figure 6, Figure 7, and Figure 10. Tables S10 and S11: IC50 values of free and encapsulated forms of Cholosens for HeLa, NHDF, S. aureus, and E. Coli.

## References

[1] Cheng L., Wang C., Liu Z. (2014). Functional nanomaterials for phototherapies of cancer. Chin. J. Clin. Oncol..

[2] Dougherty T.J., Gomer C.J., Henderson B.W., Jori G., Kessel D., Korbelik M., Moan J., Peng Q. (1998). Photodynamic therapy. J. Natl. Cancer Inst..

[3] Allison R.R., Sibata C.H. (2010). Oncologic photodynamic therapy photosensitizers: A clinical review. Photodiagnosis Photodyn. Ther..

[4] DeRosa M.C., Crutchley R.J. (2002). Photosensitized singlet oxygen and its applications. Coord. Chem. Rev..

[5] Dolmans D.E.J.G.J., Fukumura D., Jain R.K. (2003). Photodynamic therapy for cancer. Nat. Rev. Cancer.

[6] Svenskaya Y.I., Pavlov A.M., Gorin D.A., Gould D.J., Parakhonskiy B.V., Sukhorukov G.B. (2016). Photodynamic therapy platform based on localized delivery of photosensitizer by vaterite submicron particles. Colloids Surf. B Biointerfaces.

[7] Paszko E., Ehrhardt C., Senge M.O., Kelleher D.P., Reynolds J.V. (2011). Nanodrug applications in photodynamic therapy. Photodiagnosis Photodyn. Ther..

[8] Chatterjee D.K., Fong L.S., Zhang Y. (2008). Nanoparticles in photodynamic therapy: An emerging paradigm. Adv. Drug Deliv. Rev..

[9] Allison R.R. (2009). Future PDT. Photodiagnosis Photodyn. Ther..

[10] Han Y., Bu J., Zhang Y., Tong W., Gao C. (2012). Encapsulation of Photosensitizer into Multilayer Microcapsules by Combination of Spontaneous Deposition and Heat-Induced Shrinkage for Photodynamic Therapy. Macromol. Biosci..

[11] Hong E.J., Choi D.G., Shim M.S. (2016). Targeted and effective photodynamic therapy for cancer using functionalized nanomaterials. Acta Pharm. Sin. B.

[12] Chen W.H., Luo G.F., Qiu W.X., Lei Q., Liu L.H., Wang S.B., Zhang X.Z. (2017). Mesoporous silica-based versatile theranostic nanoplatform constructed by layer-by-layer assembly for excellent photodynamic/chemo therapy. Biomaterials.

[13] Ellahioui Y., Patra M., Mari C., Kaabi R., Karges J., Gasser G., Gómez-Ruiz S. (2019). Mesoporous silica nanoparticles functionalised with a photoactive ruthenium(ii) complex: Exploring the formulation of a metal-based photodynamic therapy photosensitiser. Dalt. Trans..

[14] De Koker S., De Geest B.G., Cuvelier C., Ferdinande L., Deckers W., Hennink W.E., De Smedt S.C., Mertens N. (2007). In vivo Cellular Uptake, Degradation, and Biocompatibility of Polyelectrolyte Microcapsules. Adv. Funct. Mater..

[15] Lomova M.V., Brichkina A.I., Kiryukhin M.V., Vasina E.N., Pavlov A.M., Gorin D.A., Sukhorukov G.B., Antipina M.N. (2015). Multilayer Capsules of Bovine Serum Albumin and Tannic Acid for Controlled Release by Enzymatic Degradation. ACS Appl. Mater. Interfaces.

[16] Itoh Y., Matsusaki M., Kida T., Akashi M. (2008). Locally Controlled Release of Basic Fibroblast Growth Factor from Multilayered Capsules. Biomacromolecules.

[17] De Cock L.J., De Wever O., Van Vlierberghe S., Vanderleyden E., Dubruel P., De Vos F., Vervaet C., Remon J.P., De Geest B.G. (2012). Engineered (hep/pARG)_2_polyelectrolyte capsules for sustained release of bioactive TGF-β1. Soft Matter.

[18] She Z., Wang C., Li J., Sukhorukov G.B., Antipina M.N. (2012). Encapsulation of Basic Fibroblast Growth Factor by Polyelectrolyte Multilayer Microcapsules and Its Controlled Release for Enhancing Cell Proliferation. Biomacromolecules.

[19] De Geest B.G., Willart M.A., Hammad H., Lambrecht B.N., Pollard C., Bogaert P., De Filette M., Saelens X., Vervaet C., Remon J.P. (2012). Polymeric multilayer capsule-mediated vaccination induces protective immunity against cancer and viral infection. ACS Nano.

[20] Kilic E., Novoselova M.V., Lim S.H., Pyataev N.A., Pinyaev S.I., Kulikov O.A., Sindeeva O.A., Mayorova O.A., Murney R., Antipina M.N. (2017). Formulation for Oral Delivery of Lactoferrin Based on Bovine Serum Albumin and Tannic Acid Multilayer Microcapsules. Sci. Rep..

[21] Tiourina O.P., Antipov A.A., Sukhorukov G.B., Larionova N.I., Lvov Y., Möhwald H. (2001). Entrapment of Alfa-Chymotrypsin into Hollow Polyelectrolyte Microcapsules. Macromol. Biosci..

[22] Lvov Y., Antipov A.A., Mamedov A., Möhwald H., Sukhorukov G.B. (2001). Urease Encapsulation in Nanoorganized Microshells. Nano Lett..

[23] Kreft O., Skirtach A.G., Sukhorukov G.B., Möhwald H. (2007). Remote Control of Bioreactions in Multicompartment Capsules. Adv. Mater..

[24] Antipina M.N., Kiryukhin M.V., Chong K., Low H.Y., Sukhorukov G.B. (2009). Patterned microcontainers as novel functional elements for microTAS and LOC. Lab Chip.

[25] Zelikin A.N., Becker A.L., Johnston A.P.R., Wark K.L., Turatti F., Caruso F. (2007). A General Approach for DNA Encapsulation in Degradable Polymer Microcapsules. ACS Nano.

[26] Reibetanz U., Claus C., Typlt E., Hofmann J., Donath E. (2006). Defoliation and Plasmid Delivery with Layer-by-Layer Coated Colloids. Macromol. Biosci..

[27] Kakran M., Muratani M., Tng W.J., Liang H., Trushina D.B., Sukhorukov G.B., Ng H.H., Antipina M.N. (2015). Layered polymeric capsules inhibiting the activity of RNases for intracellular delivery of messenger RNA. J. Mater. Chem. B.

[28] Tarakanchikova Y., Alzubi J., Pennucci V., Follo M., Kochergin B., Muslimov A., Skovorodkin I., Vainio S., Antipina M.N., Atkin V. (2020). Biodegradable Nanocarriers Resembling Extracellular Vesicles Deliver Genetic Material with the Highest Efficiency to Various Cell Types. Small.

[29] Timin A.S., Muslimov A.R., Petrova A.V., Lepik K.V., Okilova M.V., Vasin A.V., Afanasyev B.V., Sukhorukov G.B. (2017). Hybrid inorganic-organic capsules for efficient intracellular delivery of novel siRNAs against influenza A (H1N1) virus infection. Sci. Rep..

[30] Trushina D.B., Akasov R.A., Khovankina A.V., Borodina T.N., Bukreeva T.V., Markvicheva E.A. (2019). Doxorubicin-loaded biodegradable capsules: Temperature induced shrinking and study of cytotoxicity in vitro. J. Mol. Liq..

[31] Novoselova M.V., Loh H.M., Trushina D.B., Ketkar A., Abakumova T.O., Zatsepin T.S., Kakran M., Brzozowska A.M., Lau H.H., Gorin D.A. (2020). Biodegradable Polymeric Multilayer Capsules for Therapy of Lung Cancer. ACS Appl. Mater. Interfaces.

[32] Antipina M.N., Sukhorukov G.B. (2011). Remote control over guidance and release properties of composite polyelectrolyte based capsules. Adv. Drug Deliv. Rev..

[33] Antipina M.N., Kiryukhin M.V., Skirtach A.G., Sukhorukov G.B. (2014). Micropackaging via layer-by-layer assembly: Microcapsules and microchamber arrays. Int. Mater. Rev..

[34] Parakhonskiy B.V., Yashchenok A.M., Konrad M., Skirtach A.G. (2014). Colloidal micro- and nano-particles as templates for polyelectrolyte multilayer capsules. Adv. Colloid Interface Sci..

[35] Trushina D.B., Bukreeva T.V., Antipina M.N. (2016). Size-Controlled Synthesis of Vaterite Calcium Carbonate by the Mixing Method: Aiming for Nanosized Particles. Cryst. Growth Des..

[36] Trushina D.B., Bukreeva T.V., Borodina T.N., Belova D.D., Belyakov S., Antipina M.N. (2018). Heat-driven size reduction of biodegradable polyelectrolyte multilayer hollow capsules assembled on CaCO_3_ template. Colloids Surf. B Biointerfaces.

[37] De Cock L.J., De Koker S., De Geest B.G., Grooten J., Vervaet C., Remon J.P., Sukhorukov G.B., Antipina M.N. (2010). Polymeric Multilayer Capsules in Drug Delivery. Angew. Chem. Int. Ed..

[38] De Koker S., Hoogenboom R., De Geest B.G. (2012). Polymeric multilayer capsules for drug delivery. Chem. Soc. Rev..

[39] Antipov A.A., Sukhorukov G.B., Leporatti S., Radtchenko I.L., Donath E., Möhwald H. (2002). Polyelectrolyte multilayer capsule permeability control. Colloids Surf. A Physicochem. Eng. Asp..

[40] Mauser T., Dejugnat C., Sukhorukov G.B. (2004). Reversible pH-dependent properties of multilayer microcapsules made of weak polyelectrolytes. Macromol. Rapid Commun..

[41] Mauser T. (2006). Multilayer Capsules with Stimuli-Sensitive Properties: pH-Response and Carbohydrate-Sensing. Ph.D. Thesis.

[42] Köhler K., Shchukin D.G., Möhwald H., Sukhorukov G.B. (2005). Thermal Behavior of Polyelectrolyte Multilayer Microcapsules. 1. The Effect of Odd and Even Layer Number. J. Phys. Chem. B.

[43] Köhler K., Möhwald H., Sukhorukov G.B. (2006). Thermal Behavior of Polyelectrolyte Multilayer Microcapsules: 2. Insight into Molecular Mechanisms for the PDADMAC/PSS System. J. Phys. Chem. B.

[44] Ibarz G., Dähne L., Donath E., Möhwald H. (2002). Controlled Permeability of Polyelectrolyte Capsules via Defined Annealing. Chem. Mater..

[45] Sukhorukov G.B., Donat E.H., Moya S. (2000). Microencapsulation by means of step-wise adsorption of polyelectrolytes. J. Microencapsul..

[46] Ermakov A.V., Inozemtseva O.A., Gorin D.A., Sukhorukov G.B., Belyakov S., Antipina M.N. (2019). Influence of Heat Treatment on Loading of Polymeric Multilayer Microcapsules with Rhodamine B. Macromol. Rapid Commun..

[47] Schlenoff J.B., Ly H., Li M. (1998). Charge and Mass Balance in Polyelectrolyte Multilayers. J. Am. Chem. Soc..

[48] Jori G. (2006). Photodynamic therapy of microbial infections: State of the art and perspectives. J. Environ. Pathol. Toxicol. Oncol..

[49] Kuznetsova A.A., Lukyanets E.A., Solovyeva L.I., Knorre D.G., Fedorova O.S. (2008). DNA-binding and oxidative properties of cationic phthalocyanines and their dimeric complexes with anionic phthalocyanines covalently linked to oligonucleotides. J. Biomol. Struct. Dyn..

[50] Pashkovskaya A.A., Maizlish V.E., Shaposhnikov G.P., Kotova E.A., Antonenko Y.N. (2008). Role of electrostatics in the binding of charged metallophthalocyanines to neutral and charged phospholipid membranes. Biochim. Biophys. Acta Biomembr..

[51] Liu W., Jensen T.J., Fronczek F.R., Hammer R.P., Smith K.M., Vicente M.G.H. (2005). Synthesis and cellular studies of nonaggregated water-soluble phthalocyanines. J. Med. Chem..

[52] Calzavara-Pinton P.G., Venturini M., Sala R. (2007). Photodynamic therapy: Update 2006 part 2: Clinical results. J. Eur. Acad. Dermatol. Venereol..

[53] Makarov D.A., Yuzhakova O.A., Slivka L.K., Kuznetsova N.A., Negrimovsky V.M., Kaliya O.L., Lukyanets E.A. (2007). Cationic Zn and Al phthalocyanines: Synthesis, spectroscopy and photosensitizing properties. J. Porphyr. Phthalocyanines.

[54] Ben-Hur E., Carmichael A., Riesz P., Rosenthal I. (1985). Photochemical generation of superoxide radical and the cytotoxicity of phthalocyanines. Int. J. Radiat. Biol..

[55] Brilkina A.A., Dubasova L.V., Sergeeva E.A., Pospelov A.J., Shilyagina N.Y., Shakhova N.M., Balalaeva I.V. (2019). Photobiological properties of phthalocyanine photosensitizers Photosens, Holosens and Phthalosens: A comparative in vitro analysis. J. Photochem. Photobiol. B Biol..

[56] Kastl L., Sasse D., Wulf V., Hartmann R., Mircheski J., Ranke C., Carregal-Romero S., Martínez-López J.A., Fernández-Chacón R., Parak W.J. (2013). Multiple internalization pathways of polyelectrolyte multilayer capsules into mammalian cells. ACS Nano.

[57] Kantner K., Rejman J., Kraft K.V.L., Soliman M.G., Zyuzin M.V., Escudero A., del Pino P., Parak W.J. (2018). Laterally and Temporally Controlled Intracellular Staining by Light-Triggered Release of Encapsulated Fluorescent Markers. Chem. A Eur. J..

[58] Sumbayev V.V., Yasinska I.M. (2005). Regulation of MAP kinase-dependent apoptotic pathway: Implication of reactive oxygen and nitrogen species. Arch. Biochem. Biophys..

[59] Bauer M.K.A., Vogt M., Los M., Siegel J., Wesselborg S., Schulze-Osthoff K. (1998). Role of reactive oxygen intermediates in activation-induced CD95 (APO- 1/Fas) ligand expression. J. Biol. Chem..

[60] Roos W.P., Kaina B. (2006). DNA damage-induced cell death by apoptosis. Trends Mol. Med..

[61] Ott M., Gogvadze V., Orrenius S., Zhivotovsky B. (2007). Mitochondria, oxidative stress and cell death. Apoptosis.

[62] Green D.R. (2004). The Pathophysiology of Mitochondrial Cell Death. Science.

[63] Zong W.X., Thompson C.B. (2006). Necrotic death as a cell fate. Genes Dev..

[64] Nuutila J., Lilius E.M. (2005). Flow cytometric quantitative determination of ingestion by phagocytes needs the distinguishing of overlapping populations of binding and ingesting cells. Cytom. Part A.

