# Supplementary material for: In Vitro Bioeffects of Polyelectrolyte Multilayer Microcapsules Post-Loaded with Water-Soluble Cationic Photosensitizer
Source: Pharmaceutics. 2020 Jun 30;12(7):610. doi: 10.3390/pharmaceutics12070610 (PMC7408512; doi:10.3390/pharmaceutics12070610)
Supplement: Supplementary file 1 [file pharmaceutics-12-00610-s001.pdf]

Supplementary Materials: In Vitro Bioeffect of Polyelectrolyte Multilayer Microcapsules Post-Loaded with Water-Soluble Cationic Photosensitizer

Alexey V. Ermakov, Roman A. Verkhovskii, Irina V. Babushkina, Daria B. Trushina, Olga A. Inozemtseva, Evgeny A. Lukyanets, Vladimir J. Ulyanov, Dmitry A. Gorin, Sergei Belyakov and Maria N. Antipina

1. Temperature-induced morphological changes in the [DS/PAgr]$_4$ PMC system

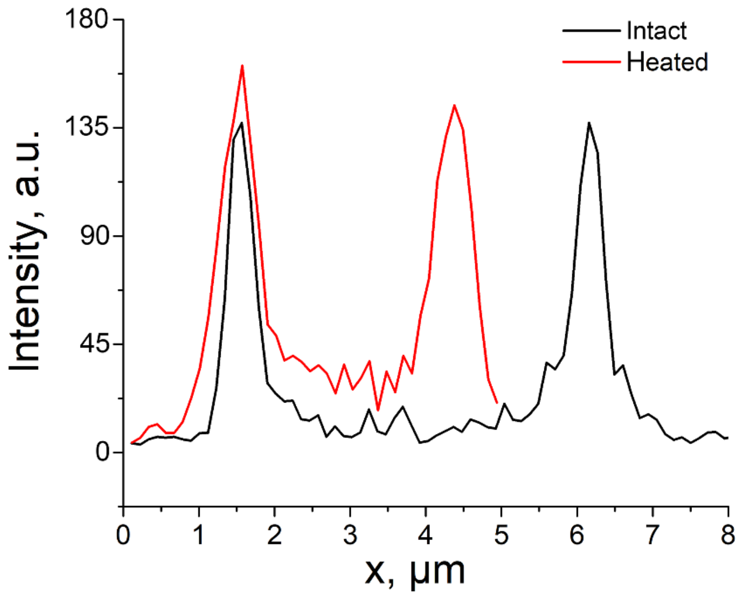

Figure S1. Fluorescence intensity cross-section profiles of the intact and heated capsules labeled with RhD6G (λ_{ex}=525 nm, λ_{em}=548 nm). The corresponding image was captured on the focal plane, allowing to visualize the maximum distance between two antipodal points on their surfaces. The capsule diameter is measured as a distance between the positions of maximums of two peaks.

2. Optical properties of Cholosens

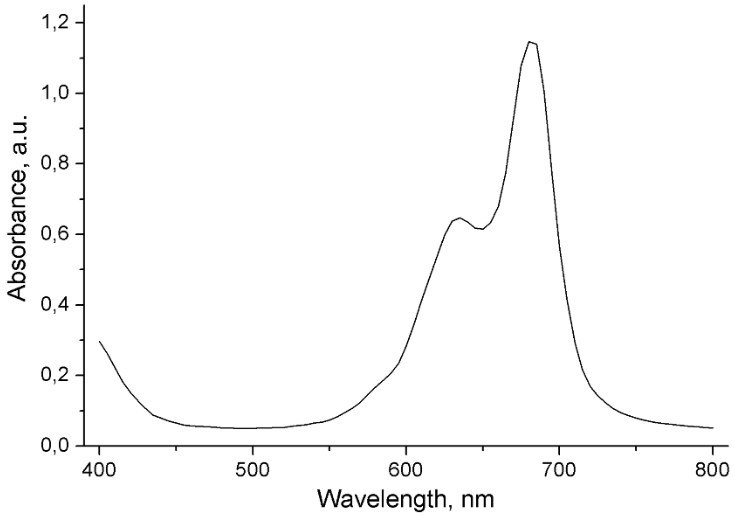

Figure S2. The absorbance spectrum of Cholosens. The spectrum was obtained in Di water (18.2 MΩcm-1) at pH 6.6. Before measurements, the capsules were thoroughly washed via three sequential centrifugation steps, each followed by the exchanging of the supernatant.

3. Loading of Cholosens in PMC: loading data based on the analysis of enzymatically degraded capsules.

Samples of lyophilized Cholosens-loaded capsules loaded as displayed in Figure 3 were each mixed with 1 mL of 1 mg/mL α-chymotrypsin dissolved in 1× PBS (pH 7.4) in 2 mL centrifuge tubes and kept at 37 C for 24 hours. Undissolved polymeric complexes were then extracted by centrifugation and resuspended again in the enzyme solution. The respective supernatants were then analyzed with fluorescence spectroscopy to determine the amount of Cholosens released from the capsules after the complete digestion of the PMC. The respective measured released data and calculated loading of Cholosens per a single capsule are shown in Figure S3.

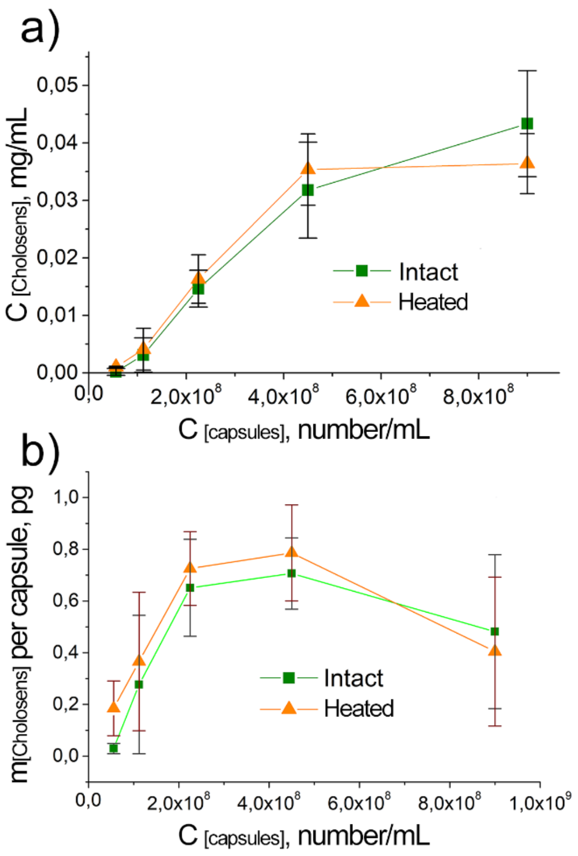

Figure S3. Loading of Cholosens in [DS/PArg]$_4$ microcapsules with and without heat treatment (80 °C, 60 min) depending on the capsule concentration: (**a**) spectroscopically measured concentration of Cholosens in the supernatant after the capsule enzymatic degradation; (**b**) calculated amount of Cholosens in pg released from a single capsule.

3. Student's test results

3.1. Student's test results for the cellular uptake of [DS/PArg]$_4$ microcapsules by HeLa cells (Figure 6 ***a****)*

The number of measurements was 3 for all uptake experiments; a p-value < 0.05 was considered statistically significant. Determination of the statistical significance of the differences between cellular uptake of the intact and heated PMC is presented in Table S1.

Table S1. Flow cytometry data on the capsules internalization by HeLa cells and calculated t parameter (n.s. means non-significant).

Time of incubation, h	Cells with Intact microcapsulesules, %	Cells with Heated microcapsulesules, %	t	degrees of freedom, df	t crit (* p<0.05)	result
1	32±3.5	42.6±4.4	3.2655	4	2.7764	*
2	41.5±5.2	52±3	3.0294			*
4	55±6	57.8±3.9	0.6777			n.s.
24	78.3±7.2	81.8±5.9	0.6512			n.s.

2.2. Student's test results for light and dark viability of HeLa and NHDF cells in the presence of free and encapsulated forms of Cholosens *(Figure 7)*

Table S2. Viability of HeLa and NHDF after treatment with 1 or 5 capsules per cell after irradiation (information for Fig.7b). Each point indicates the mean± (n=6).

HeLa, 1 capsules/cell						
**With heated capsules, irradiated**	**In Cholosens solution, irradiated**	**t**	**df**	**t crit (* p<0.05)**	**t crit (** p<0.01)**	**result**
39.76±0.77	33.94±0.72	13.5233	10	2.2281	3.1693	**
**With intact capsules, irradiated**		8.0051				**
39.26±1.46						
HeLa, 5 capsules/cell						
**With heated capsules, irradiated**	**In Cholosens solution, irradiated**	6.2897				**
10.79±2.16	4.47±1.18					
**With intact capsules, irradiated**		1.0544				n.s.
5.02±0.49						
NHDF, 1 capsules/cell						
**With heated capsules, irradiated**	**In Cholosens solution, irradiated**	14.3835				**
39.00±0.92	32.14±0.72					
**With intact capsules, irradiated**		10.3637				**
38.28±1.26						
NHDF, 5 capsules/cell						
**With heated capsules, irradiated**	**In Cholosens solution, irradiated**	12.0377				**
4.62±0.06	3.02±0.32					
**With intact capsules, irradiated**		3.2268				**
4.07±0.73						

Table S3. Viability of HeLa and NHDF after treatment with free cholosens upon laser irradiation in comparison to the non-irradiated samples. Each point indicates the mean± (n=6). Laser irradiation provides a significantly high level of toxicity in both cell lines.

HeLa							
**Cholosens dose, capsule/cell equivalent**	**In Cholosens solution, irradiated**	**In Cholosens solution, non-irradiated**	**t**	**df**	**t crit (* p<0.05)**	**t crit (** p<0.01)**	**result**
1	33.94±0.72	75.30±0.65	104.4442	10	2.2281	3.1693	**
5	4.47±1.18	61.32±0.70	101.4963				**
NHDF							
**Cholosens dose, capsule/cell equivalent**	**In Cholosens solution, irradiated**	**In Cholosens solution, non-irradiated**	**t**				**result**
1	32.14±0.72	77.25±0.73	107.7668				**
5	3.02±0.32	67.54±0.68	210.2919				**

Table S4. Viability of HeLa and NHDF cell lines treated with Cholosens-loaded intact capsules with and without laser irradiation.

HeLa							
**Cholosens dose, capsules/cell**	**With intact capsules, irradiated**	**Win intact capsules, non-irradiated**	**t**	**df**	**t crit (* p<0.05)**	**t crit (** p<0.01)**	**result**
1	39.26±1.46	99.78±2.15	57.0415	10	2.2281	3.1693	**
5	5.02±0.49	105.78±2.55	95.0496				**
20	2.88±0.08	108.92±15.41	16.8553				**
40	3.03±0.53	104.84±6.08	40.8619				**
80	2.92±0.28	104.76±8.17	30.5153				**
NHDF							
**Cholosens dose, capsules/cell**	**With intact capsules, irradiated**	**Win intact capsules, non-irradiated**	**t**				
1	38.28±1.26	89.87±2.86	40.4349				**
5	4.07±0.73	79.57±3.46	52.2985				**
20	2.54±0.31	42.33±6.49	15.0006				**
40	2.50±0.35	38.16±9.69	9.0085				**
80	2.45±0.05	38.13±2.43	35.9586				**

Table S5. Viability of HeLa and NHDF cells after treatment with both intact and heated PMC loaded with Cholosens. Each point indicates the mean± (n=6).

HeLa, 1 capsules/cell						
**With heated capsules, irradiated**	**With intact capsules, irradiated**	**t**	**df**	**t crit (* p<0.05)**	**t crit (** p<0.01)**	**result**
39.76±0.77	39.26±1.46	0.7420	10	2.2281	3.1693	n.s.
**With heated capsules, non-irradiated**	**With intact capsules, non-irradiated**	5.2678				**
93.18±2.19	99.78±2.15					
HeLa, 5 capsules/cell						
**With heated capsules, irradiated**	**With intact capsules, irradiated**	**t**				**
10.79±2.16	5.02±0.49	6.3812				
**With heated capsules, non-irradiated**	**With intact capsules, non-irradiated**	10.9701				**
89.18±2.69	105.78±2.55					
HeLa, 20 capsules/cell						
**With heated capsules, irradiated**	**With intact capsules, irradiated**	**t**				**

2.58±0.10	2.88±0.08	5.7382				
**With heated capsules, non-irradiated**	**With intact capsules, non-irradiated**	0.2968				n.s.
111.10±9.28	108.92±15.41					
HeLa, 40 capsules/cell						
**With heated capsules, irradiated**	**With intact capsules, irradiated**	**t**				n.s.
2.86±0.04	3.03±0.53	0.7835				
**With heated capsules, non-irradiated**	**With intact capsules, non-irradiated**	1.9649				n.s.
95.90±9.34	104.84±6.08					
HeLa, 80 capsules/cell						
**With heated capsules, irradiated**	**With intact capsules, irradiated**	**t**				n.s.
2.99±0.12	2.92±0.28	0.5629				
**With heated capsules, non-irradiated**	**With intact capsules, non-irradiated**	0.9140				n.s.
107.82±0.71	104.76±8.17					
NHDF, 1 capsules/cell						
**With heated capsules, irradiated**	**With intact capsules, irradiated**	**t**	**df**			n.s.
39,00±0,92	38,28±1,26	1.1304	10			
**With heated capsules, non-irradiated**	**With intact capsules, non-irradiated**	1.0333				n.s.
87,96±3,51	89,87±2,86					
NHDF, 5 capsules/cell						
**With heated capsules, irradiated**	**With intact capsules, irradiated**	**t**	**df**			n.s.
4,62±0,06	4,07±0,73	1.8393	10			
**With heated capsules, non-irradiated**	**With intact capsules, non-irradiated**	2.5310				*
65,16±13,51	79,57±3,46					
NHDF, 20 capsules/cell						
**With heated capsules, irradiated**	**With intact capsules, irradiated**	**t**	**df**			n.s.
2,52±0,40	2,54±0,31	0.0968	10			
**With heated capsules, non-irradiated**	**With intact capsules, non-irradiated**	3.2666				**
66,51±16,93	42,33±6,49					
NHDF, 40 capsules/cell						
**With heated capsules, irradiated**	**With intact capsules, irradiated**	**t**	**df**			n.s.
2,43±0,03	2,50±0,35	0.4881	10			
**With heated capsules, non-irradiated**	**With intact capsules, non-irradiated**	1.1925				n.s.
33,06±3,98	38,16±9,69					
NHDF, 80 capsules/cell						
**With heated capsules, irradiated**	**With intact capsules, irradiated**	**t**	**df**			**
2,69±0,16	2,45±0,05	3.5070	10			
**With heated capsules, non-irradiated**	**With intact capsules, non-irradiated**	0.6624				n.s.
39.30±3.58	38.13±2.43					

Table S6. Viability of non-irradiated HeLa cells treated with 1 or 5 capsules per cell (for Figure 7b). Each point indicates the mean± (n=6). Both intact and heated Cholosens loaded PMC displayed significantly lower non-irradiated toxicity to HeLa cells compared to Cholosens solution (p<0.01).

HeLa, 1 capsules/cell						
**With heated capsules, non-irradiated**	**In Cholosens solution, non-irradiated**	**t**	**df**	**t crit (* p<0.05)**	**t crit (** p<0.01)**	**result**
93,18±2,19	75,30±0,65	19.1719	10	2,2281	3,1693	**
**With intact capsules, non-irradiated**	**In Cholosens solution, non-irradiated**	26.6966				**
99,78±2,15	75,30±0,65					
HeLa, 5 capsules/cell						
**With heated capsules, non-irradiated**	**In Cholosens solution, non-irradiated**					**
89,18±2,69	61,32±0,70	24.5514				
**With intact capsules, non-irradiated**	**In Cholosens solution, non-irradiated**	41.1840				**
105.78±2.55	61.32±0.70					

2.3. Student's test results for light and dark viability of bacterial cells depending on the number of Cholosens-loaded capsules (intact or heated) added to the culture medium

Table S7. Viability of gram-positive *S. aureus* bacterial cells, %. Each point indicates the mean± (n=6).

3 capsules/cell						
**With heated capsules, irradiated**	**With intact capsules, irradiated**	**t**	**df**	**t crit (* p<0.05)**	**t crit (** p<0.01)**	**result**
37.5±3.115	25±3.129	6.9348	10	2.2281	3.1693	**
**In Cholosens solution, irradiated**	**With intact capsules, irradiated**					**
7±3,115	25±3,129	9.9862				
**In Cholosens solution, irradiated**	**With heated capsules, irradiated**					**
7±3.115	37.5±3.115	16.9591				
**With heated capsules, non-irradiated**	**With intact capsules, non-irradiated**					**
82±4.115	69.7±6.115	4.0877				
**In Cholosens solution, non-irradiated**	**With intact capsules, non-irradiated**					**
29±3.115	69.7±6.115	14.5270				
**In Cholosens solution, non-irradiated**	**With heated capsules, non-irradiated**					**
29±3.115	82±4.115	25.1544				
9 capsules/cell						
**With heated capsules, irradiated**	**With intact capsules, irradiated**	**t**				
7.8±2.264	5.6±2.078	1.7536				n.s.
**In Cholosens solution, irradiated**	**With intact capsules, irradiated**					
3.05±1.864	5.6±2.078	2.2376				*
**In Cholosens solution, irradiated**	**With heated capsules, irradiated**					
3.05±1.864	7.8±2.264	3.9675				**
**With heated capsules, non-irradiated**	**With intact capsules, non-irradiated**					
63±3.864	51±3.864	5.3790				**
**In Cholosens solution, non-irradiated**	**With intact capsules, non-irradiated**					
11±2.164	51±3.864	17.9301				**
**In Cholosens solution, non-irradiated**	**With heated capsules, non-irradiated**					

11±2.164	63±3.864	28.7609				**
18 capsules/cell						
**With heated capsules, irradiated**	**With intact capsules, irradiated**	**t**				
5.75±1.6675	4.25±2.1825	1.3377				n.s.
**In Cholosens solution, irradiated**	**With intact capsules, irradiated**					
1.875±1.0175	4.25±2.1825	2.4159				*
**In Cholosens solution, irradiated**	**With heated capsules, irradiated**					
1.875±1.0175	5.75±1.6675	4.8590				**
**With heated capsules, non-irradiated**	**With intact capsules, non-irradiated**					
51±3.1675	44±3.1675	3.8277				**
**In Cholosens solution, non-irradiated**	**With intact capsules, non-irradiated**					
6±0.99	44±3.1675	28.0481				**
**In Cholosens solution, non-irradiated**	**With heated capsules, non-irradiated**					
6±0.99	51±3.1675	33.2148				**
27 capsules/cell						
**With heated capsules, irradiated**	**With intact capsules, irradiated**	**t**				
1.6±0.898	0±1.31	2.4676				*
**In Cholosens solution, irradiated**	**With intact capsules, irradiated**					
0±1.298	0±1.31	0				n.s.
**In Cholosens solution, irradiated**	**With heated capsules, irradiated**					
0±1.298	1.6±0.898	2.4831				*
**With heated capsules, non-irradiated**	**With intact capsules, non-irradiated**					
43±3.898	33±4.398	4.1681				**
**In Cholosens solution, non-irradiated**	**With intact capsules, non-irradiated**					
0±0.898	33±4.398	18.0080				**
**In Cholosens solution, non-irradiated**	**With heated capsules, non-irradiated**					
0±0.898	43±3.898	26.3313				**

Table S8. Viability of gram-negative E. coli., %. Each point indicates the mean± (n=6).

5 capsules/cell						
**With heated capsules, irradiated**	**With intact capsules, irradiated**	**t**	**df**	**t crit (* p<0.05)**	**t crit (** p<0.01)**	**result**
81.65066±5.08253	68.39813±5.08253	4.5163	10	2.2281	3.1693	**
**In Cholosens solution, irradiated**	**With intact capsules, irradiated**					
39.22372±5.08253	68.39813±5.08253	9.9422				**
**In Cholosens solution, irradiated**	**With heated capsules, irradiated**					
39.22372±5.08253	81.65066±5.08253	14.4585				**
**With heated capsules, non-irradiated**	**With intact capsules, non-irradiated**					
95.06878±3.08253	93.49001±4.08253	0.7560				n.s.
**In Cholosens solution, non-irradiated**	**With intact capsules, non-irradiated**					
77.36143±4.08253	93.49001±4.08253	6.8427				**
**In Cholosens solution, non-irradiated**	**With heated capsules, non-irradiated**					
77.36143±4.08253	95.06878±3.08253	8.4788				**
10 capsules/cell						
**With heated capsules,**	**With intact capsules,**	**t**				

**irradiated**	**irradiated**					
72.22943±4.08253	54.58033±6.08253	5.9014				**
**In Cholosens solution, irradiated**	**With intact capsules, irradiated**					
34.82715±4.08253	54.58033±6.08253	6.6050				**
**In Cholosens solution, irradiated**	**With heated capsules, irradiated**					
34.82715±4.08253	72.22943±4.08253	15.8683				**
**With heated capsules, non-irradiated**	**With intact capsules, non-irradiated**					
91.98547±4.08253	86.29847±4.08253	2.4128				*
**In Cholosens solution, non-irradiated**	**With intact capsules, non-irradiated**					
67.70496±4.61147	86.29847±4.08253	7.3949				**
**In Cholosens solution, non-irradiated**	**With heated capsules, non-irradiated**					
67.70496±4.61147	91.98547±4.08253	9.6567				**
20 capsules/cell						
**With heated capsules, irradiated**	**With intact capsules, irradiated**	**t**				**result**
47.62523±6.08253	32.59798±5.58253	4.4585				**
**In Cholosens solution, irradiated**	**With intact capsules, irradiated**					
25.66831±5.08253	32.59798±5.58253	2.2483				*
**Cholosens solution irradiated**	**With heated capsules, irradiated**					
25.66831±5.08253	47.62523±6.08253	6.7853				**
**With heated capsules, non-irradiated**	**With intact capsules, non-irradiated**					
84.26291±6.08253	59.43421±6.08253	7.0702				**
**In Cholosens solution, non-irradiated**	**With intact capsules, non-irradiated**					
51.49234±6.38126	59.43421±6.08253	2.2067				n.s.
**In Cholosens solution, non-irradiated**	**With heated capsules, non-irradiated**					
51.49234±6.38126	84.26291±6.08253	9.1054				**
80 capsules/cell						
**With heated capsules, irradiated**	**With intact capsules, irradiated**	**t**				
28.15988±5.425	3.3132±1.11601	10.4210				**
**In Cholosens solution, irradiated**	**With intact capsules, irradiated**					
0.59694±4.08253	1.11601±3.3132	0.2418				n.s.
**Cholosens solution irradiated**	**With heated capsules, irradiated**					
0.59694±4.08253	28.15988±5.425	9.9440				**
**With heated capsules, non-irradiated**	**With intact capsules, non-irradiated**					
87.28108±8.08253	58.13652±8.08253	6.2456				**
**In Cholosens solution, non-irradiated**	**With intact capsules, non-irradiated**					

51.1809±7.311	58.13652±8.08253	1.5633				n.s.
**In Cholosens solution, non-irradiated**	**With heated capsules, non-irradiated**					
51.1809±7.311	87.28108±8.0825	8.1137				**

Table S9. The difference in viability of gram-positive *S. aureus* and gram-negative *E. coli*. (%) at different doses of capsules per cell

S. aureus, With heated capsules, irradiated						
**3 capsules/cell**	**9 capsules/cell**	**t**	**df**	**t crit (* p<0.05)**	**t crit (** p<0.01)**	**result**
37.5±3.115	7.8±2.264	18.8920	10	2,2281	3,1693	**
S. aureus, With heated capsules, non-irradiated						
**3 capsules/cell**	**9 capsules/cell**					
82±4.115	63±3.864	8.2448				**
S. aureus, With intact capsules, irradiated						
**3 capsules/cell**	**9 capsules/cell**					
25±3.129	5.6±2.078	12.6513				**
S. aureus, With intact capsules, non-irradiated						
**3 capsules/cell**	**9 capsules/cell**					
69.7±6.115	51±3.864	6.3324				**
S. aureus, in Cholosens solution, irradiated						
**3 capsules/cell**	**9 capsules/cell**					
7±3.115	3.05±1.864	2.6653				*
S. aureus, in Cholosens solution, non-irradiated						
**3 capsules/cell**	**9 capsules/cell**					
29±3.115	11±2.164	11.6246				**
E. coli., with heated capsules, irradiated						
**10 capsules/cell**	**20 capsules/cell**	**t**				
72.22943±4.08253	47.62523±6.08253	8.2270				**
E. coli., with heated capsules, non-irradiated						
**10 capsules/cell**	**20 capsules/cell**					
91.98547±4.08253	84.26291±6.08253	2.5822				*
E. coli., with intact capsules, irradiated						
**10 capsules/cell**	**20 capsules/cell**					
54.58033±6.08253	32.59798±5.58253	6.5220				**
E. coli., with intact capsules, non-irradiated						
**10 capsules/cell**	**20 capsules/cell**					
86.29847±4.08253	59.43421±6.08253	8.9827				**
E. coli., in *Cholosens solution, irradiated*						
**10 capsules/cell**	**20 capsules/cell**					
34.82715±4.08253	25.66831±5.08253	3.4413				**
E. coli., in *Cholosens solution, non-irradiated*						
**10 capsules/cell**	**20 capsules/cell**					
67.70496±4.61147	51.49234±6.38126	5.0441				**

Table S10. IC50 for HeLa and NHDF cells

Irradiated						
	Intact capsules		Heated capsules		Free Cholosens	
Cell type	cap per cell	m[Cholosens], pg	cap per cell	m[Cholosens], pg	cap per cell equivalent	m[Cholosens], pg
HeLa	3.25	2.9	3.73	3.4	2.89	2.6
NHDF	3.1	2.8	3.2	2.88	2.64	2.4
Non-irradiated						
	Intact capsules		Heated capsules		Free Cholosens	
Cell type	cap per cell	m[Cholosens], pg	cap per cell	m[Cholosens], pg	cap per cell equivalent	m[Cholosens], pg
HeLa	Not established*	Not established*	Not established*	Not established*	9.1	8.2
NHDF	16,91	15,2	29.87	26.9	13.6	12.2

* The cell viability never drops below 50%

Table S11. IC50 for *S. aureus and E. Coli*

Irradiated						
	Intact capsules		Heated capsules		Free Cholosens	
Cell type	cap per cell	m[Cholosens], pg	cap per cell	m[Cholosens], pg	cap per cell equivalent	m[Cholosens], pg
S. aureus	0.85	0.77	0.94	0.846	Not established**	Not established**
E. Coli	12.2	10.8	17.1	26.9	Not established**	Not established**
Non-irradiated						
	Intact capsules		Heated capsules		Free Cholosens	
Cell type	cap per cell	m[Cholosens], pg	cap per cell	m[Cholosens], pg	cap per cell equivalent	m[Cholosens], pg
S. aureus	10.3	0.9	19.13	0.9	Not established**	Not established**
E. Coli	493***	443.7***	636***	572.4***	316***	284.4***

** The cell viability drops below 50%

*** Not practicable
